# Membrane glycerolipidome of soybean root hairs and its response to nitrogen and phosphate availability

**DOI:** 10.1038/srep36172

**Published:** 2016-11-04

**Authors:** Fang Wei, Brian Fanella, Liang Guo, Xuemin Wang

**Affiliations:** 1 Oil Crops Research Institute of Chinese Academy of Agricultural Sciences, Key Laboratory of Biology and Genetic Improvement of Oil Crops of Ministry of Agriculture, Wuhan, Hubei, 430062, China; 2 Department of Biology, University of Missouri, St. Louis, MO 63121, USA; 3 National Key Laboratory of Crop Genetic Improvement, Huazhong Agricultural University, Wuhan 430070, China; 4 Donald Danforth Plant Science Center, St. Louis, MO 63132, USA.

## Abstract

Root hairs are tubular extensions of specific root epidermal cells important in plant nutrition and water absorption. To determine membrane glycerolipids in root hairs and roots may differ, as well as their respective response to nutrient availability, this study analyzed the membrane glycerolipid species in soybean root hairs and in roots stripped of root hairs, and their response to nitrogen (N) and phosphate (P_i_) supplementation. The ratio of phospholipids to galactolipids was 1.5 fold higher in root hairs than in stripped roots. Under P_i_ deficiency, the ratio of phospholipids to galactolipids in stripped roots decreased with the greatest decrease found in the level of phosphatidylethanolamine (PE) in root hairs and stripped roots, and root hairs had an increased level of phosphatidic acid (PA). When seedlings were not supplied with N, the level of the N-containing lipids PE and phosphatidylserine in root hairs decreased whereas the level of non-N-containing lipids galactolipids and PA increased compared to N-supplied conditions. In stripped roots, the level of major membrane lipids was not different between N-sufficient and -deficient conditions. The results indicate that the membrane glycerolipidomes in root hairs are more responsive to nutrient availability than are the rest of roots.

Polar glycerolipids are major structural constituents of cellular membranes and play an important role in maintaining cellular integrity. In addition, membrane lipids are involved in mediating various cellular processes in plant growth, development, and response to environmental changes^1^,^2^. In plants, membrane glycerolipids consist of phosphoglycerolipids and non-phopshorus-containing glycolipids, such as digalactosyldiacylglycerol (DGDG) and monogalactosyldiacylglycerol (MGDG; Supplemental Fig. 1). Some of the phosphoglycerolipids also contain nitrogen (N) in their head groups, which include phosphatidylcholine (PC), phosphatidylethanolamine (PE), phosphatidylserine (PS), lysophosphatidylcholine (LPC), lysophosphatidylethanolamine (LPE), and lysophosphatidylglycerol (LPG). By comparison, phosphatidylglycerol (PG), phosphatidylinositol (PI), and phosphatidic acid (PA) are non-nitrogenous phosphoglycerolids (Supplemental Fig. 1)^3^. In addition, each head-group class of glycerolipids, such as PC, PE, and DGDG, is composed of many molecular species because two acyl groups may differ in their number of carbons and double bonds (Supplemental Fig. 1). Membrane lipid composition of a plant can differ substantially under different growth conditions. For example, under phosphate (P_i_) deprivation, the level of membrane phospholipids, such as PC, decreases whereas that of galactolipids, particularly DGDG, increases^4^,^5^,^6^. These changes divert phosphorus from phospholipids for other critical cell functions. On the other hand, in N-deprived Arabidopsis seedlings, the level of galactolipids decreased^7^. An analysis of membrane lipids of soybean showed that under N deprivation, some phosphatidylethanolamine (PE) species increased^8^. In N utilization, PE has a unique function as it is covalently conjugated to the autophagy protein ATG8. The ATG8-PE conjugation is essential to the formation of the double-membrane vesicles known as autophagosomes, critical for nutrient recycling and remobilization^9^. In addition, membrane lipids play regulatory roles in plant response to stress and nutrient availability. The hydrolysis of PE to phosphatidic acid (PA) by the phospholipase D PLDε promotes plant growth under low N availability, leading to an increase in root surface area and improved N uptake and utilization in Arabidopsis^10^ and rapeseed plants^11^. On the other hand, the hydrolysis of PC to PA by PLDζs promotes lipid remodeling and root growth under P_i_ deprivation^6^.

The current information on membrane lipid changes in plants comes primarily from lipid analyses of the whole organism or multicellular tissues, such as seedlings, leaves, or roots. These measurements might overlook the response of specific cell types that could respond strongly to nutrient availability but would be weakened by the presence of other non-responding types of cells. In particular, root hairs play an important role in increasing the plant’s ability to absorb water and nutrients. As extensions of specialized, tube-shaped epidermal cells on primary and secondary roots, root hairs increase root surface area and expand the nutrient depletion zone around the root for plant access to relatively immobile nutrients such as P_i_. Thus, root hairs can make a significant contribution to the efficiency of nutrient uptake^12^. A limiting factor for lipidomic studies of a single cell type is the difficulty of obtaining root hair cells in sufficient purity and quantity for analysis. The larger root size of soybean (*Glycine max*), which is an important crop for animal feed, oil, and biodiesel production^13^, enables isolation of sufficient quantities of root hairs used for transcriptomic, proteomic, and metabolomic analyses^14^,^15^,^16^,^17^,^18^,^19^,^20^. Therefore, this study was undertaken using soybean to analyze the membrane glycerolipidome of the single cell type, root hairs, and to explore how membrane glycerolipids change in response to nutrient availability.

## Results

### Glycerolipid Species of Root Hairs and Stripped Roots

**Figure 1:**
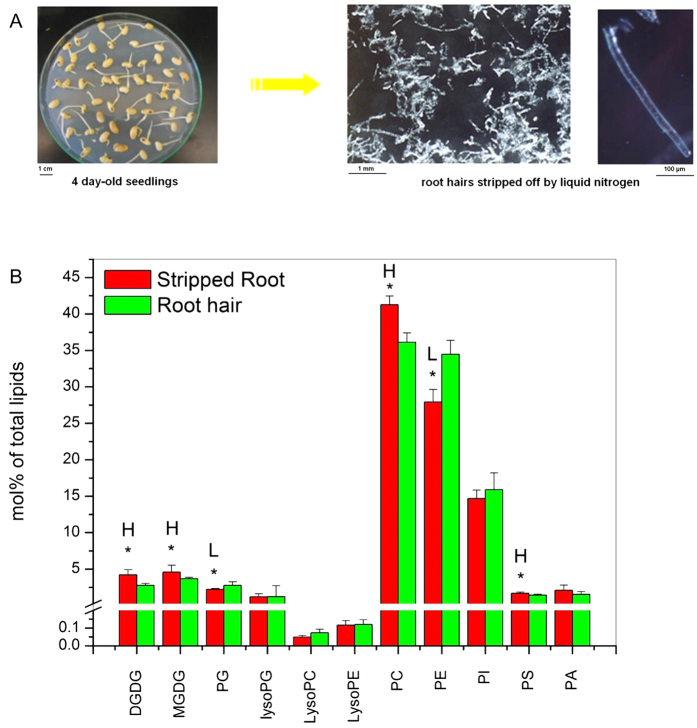
Comparison of glycerolipid classes in soybean root hairs and stripped roots. (**A**) Young soybean seedlings used for root hair isolation (*left*) and isolated root hairs (*right*). (**B**) Amounts of phospholipids and galactolipids between root hairs and stripped roots. Lipids were extracted from stripped roots and root hairs from 7-day-old seedlings grown on normal Murashige and Skoog agar medium conditions. Each glycerolipid amount is expressed as normalized mass spectral signal/total normalized glycerolipid mass spectral signal (to produce percentage of normalized MS signal, mol% of total lipids). The values are the mean ± SD (n = 10). The data of soybean stripped roots and root hairs were compared via *t* test and the P < 0.05 is indicated by *, indicating a significant difference. The value for stripped roots is higher (represented as H) or lower (represented as L) than the value for root hairs.

Soybean root hairs were isolated by stripping them from roots in liquid N^17^. High quality root hairs were collected from 100 four-day-old seedlings (Fig. 1A), yielding approximately 2.5 mg (dry weight) of root hairs that was sufficient for quantitative lipid analysis. Lipids extracted from root hairs and stripped roots (roots from which root hairs were removed) were analyzed using an electrospray ionization triple quadrupole tandem mass spectrometer (ESI-MS/MS). The data provided information on phospholipids and glycolipids speciated to the level of head group and number of carbon atoms and double bonds present in the acyl chains. The present analysis identified 140 polar plant membrane glycerolipid molecular species, including the major membrane lipid classes PC, PE, PG, PI, MGDG, and DGDG, and minor classes PA, PS, LPC, LPE, and LPG, as well as minor acyl species within each head group class (Fig. 1B).

Membrane glycerolipid species between root hairs and stripped roots were first compared, and all the lipids analyzed are numbered and presented in Supplemental Table 1, with representative structures of each lipid class shown in Supplemental Fig. 1 3. Phospholipids are the major lipids in both root hairs and stripped roots, but root hairs contain a higher level of phospholipids to galactolipid ratios than stripped roots. The level of phospholipids (mol%) in root hairs was approximately 15-fold greater than that of galactolipids whereas the phospholipid level was 10-fold greater than that in stripped roots (Fig. 1B; Supplemental Table 1). Some of the major lipid classes were different between stripped roots and root hairs. The levels (mol%) of MGDG and DGDG were 1.3- and 1.5-fold higher, respectively, in stripped roots than in root hairs, whereas the level of PG and PE were 1.2- and 1.3-fold higher, respectively, in root hairs than in stripped roots (Fig. 1B). The levels of LPG, LPC, LPE, PI and PA were comparable between stripped roots and root hairs.

**Figure 2:**
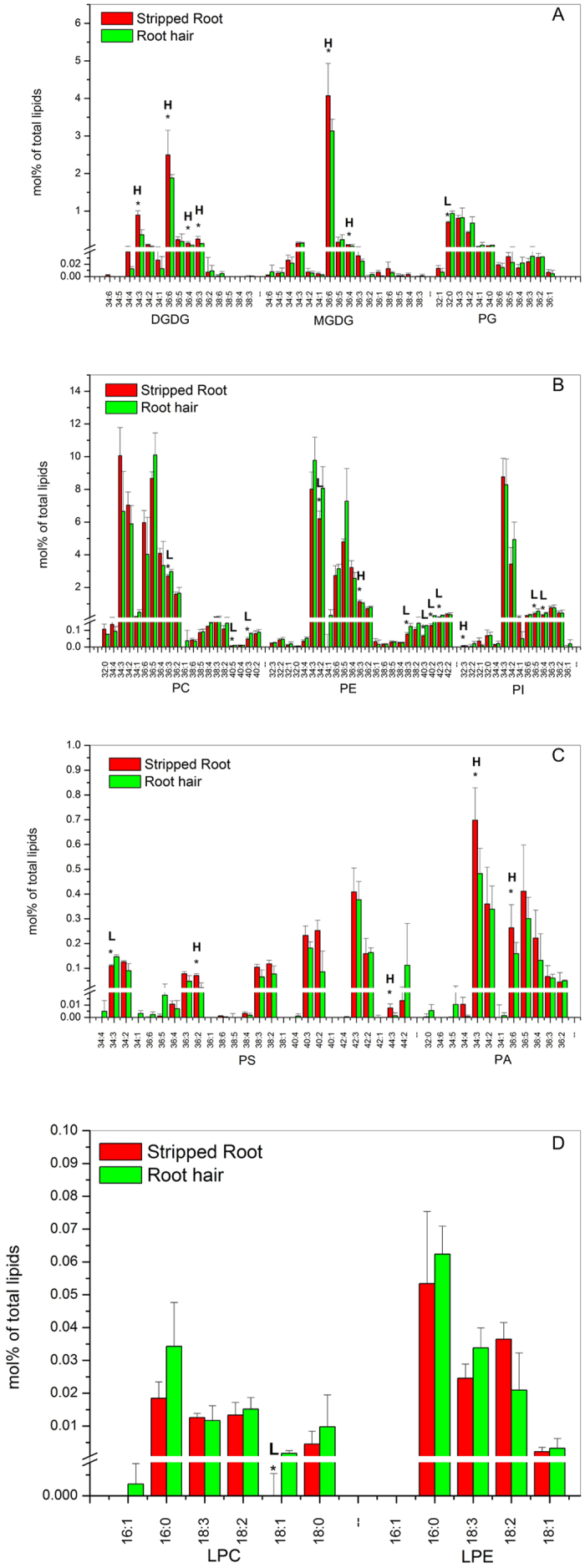
Glycerolipid molecular species in soybean root hairs and stripped roots. Lipids were extracted from stripped roots and root hairs collected from 7-day-old seedlings. Each glycerolipid molecular species is expressed as normalized mass spectral signal/total normalized glycerolipid mass spectral signal (to produce percentage of normalized MS signal, mol% of total lipids). The values are the mean ± SD (n = 10). The data of soybean stripped root and root hair were compared via *t* test and the P < 0.05 is indicated by *, indicating a significant difference. The value for stripped roots is higher (represented as H) or lower (represented as L) than the value for root hairs.

Each class of membrane glycerolipids is composed of various molecular species with varied lengths of fatty acid chains and degrees of unsaturation (Fig. 2). The highly polyunsaturated 36:6-MGDG accounts for more than 85% of MGDG whereas DGDG is composed mostly of 36:6- and 34:3-species in stripped roots and root hairs. The level of most galactolipid species, such as 34:3-DGDG, 36:6-DGDG, 36:4-DGDG, 36:3-DGDG, 36:6-MGDG and 36:4-MGDG, was lower in root hairs than in stripped roots (Fig. 2A; Supplemental Table 1). In phospholipids, root hairs had a higher level of 36:3-PC, 40:5-PC and 40:3-PC than stripped roots. In addition, compared to stripped roots, root hairs had a higher level of 32:4-PE and also a higher level of very long chain fatty acid-containing species, including 38:3-PE, 40:3-PE, 40:2-PE, and 42:3-PE. The levels of 36:5-PI and 36:4-PI were higher in root hairs than stripped roots (Fig. 2B; Supplemental Table 1). The level of 32:0-PG was higher in root hairs than in stripped roots (Fig. 2A; Supplemental Table 1). Root hairs had a lower level of 36:2-PS and 44:3-PS but a higher level of 34:3-PS than stripped roots. The levels of 34:3-PA and 36:6-PA were lower in root hairs than stripped roots (Fig. 2C; Supplemental Table 1). The levels of lysophospholipids were comparable between stripped roots and root hairs, except that root hairs were higher in 18:1-LPC than in stripped roots (Fig. 2D; Supplemental Table 1).

### Glycerolipid Changes in Root Hairs and Stripped Roots in Response to N Supply

One major function of root hairs is nutrient absorption. To study how ambient nutrient availability affects membrane glycerolipidomes, we grew seedlings on nitrogen-free agar medium^21^ solidified with 0.8% (w/v) agarose for four days. Seedlings sprayed with 10 mM of NH_4_NO_3_ or sterile water are referred to as the N-sufficient or deficient condition, respectively. After 12-hour treatments, root hairs and corresponding stripped roots were collected for lipid profiling, and all lipid species analyzed are numbered and presented in Supplemental Table 2.

No obvious difference in root and root hair morphology was observed with or without N treatment at the time of sampling.

**Figure 3:**
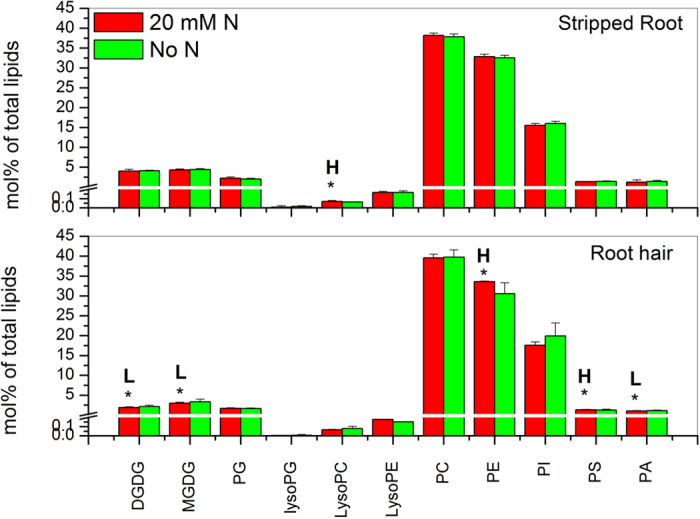
Glycerolipid classes in soybean root hairs and stripped roots with and without N supply. Four-day-old seedlings grown on nitrogen-free B&D agar medium were treated with 10 mM NH_4_NO_3_ (20 mM N) or water (No N) for 12 hours. Glycerolipid amounts are expressed as normalized mass spectral signal/total normalized glycerolipid mass spectral signal (to produce percentage of normalized MS signal, mol% of total lipids). The values are the mean ± SD (n = 10). The data of soybean stripped roots and root hairs were compared via *t* test and the P < 0.05 is indicated by *, indicating a significant difference. The value for nitrogen-sufficient seedlings is higher (represented as H) or lower (represented as L) than the value for nitrogen-deficient seedlings.

**Figure 4:**
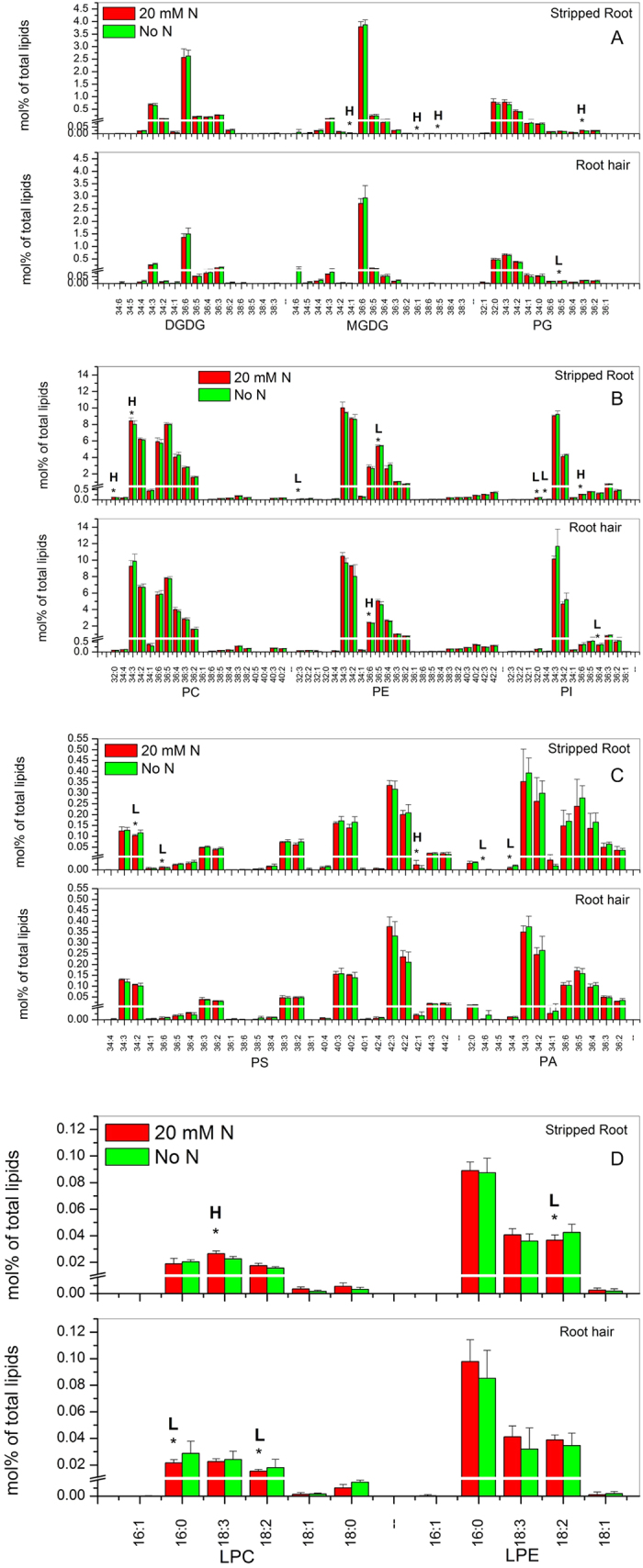
Glycerolipid molecular species in soybean root hairs and stripped roots with and without N supply. Four-day-old seedlings grown on nitrogen-free B&D agar medium were treated with 10 mM NH_4_NO_3_ (20 mM N) or water (No N) for 12 hours. Glycerolipid amounts are expressed as normalized mass spectral signal/total normalized glycerolipid mass spectral signal (to produce percentage of normalized MS signal, mol% of total lipids). The values are the mean ± SD (n = 10). The data of soybean stripped roots and root hairs were compared via *t* test and the P < 0.05 is indicated by *, indicating a significant difference. The value for nitrogen-sufficient seedlings is higher (represented as H) or lower (represented as L) than the value for nitrogen-deficient seedlings.

In root hairs, the level of DGDG, MGDG, PE, PS and PA showed a significant difference (*p* < 0.05) between N-sufficient and deficient conditions. The levels of N-containing lipids PE and PS were higher under N-sufficient than -deficient conditions, whereas the levels of non-N-containing lipids DGDG, MGDG, and PA decreased more under N-sufficient conditions than under N-deficient conditions (Fig. 3; Supplemental Table 2). The overall levels of PG, PC, and PI remained unchanged between N-sufficient and deficient conditions (Fig. 3; Supplemental Table 2), but some molecular species differed between N-sufficient and -deficient conditions. Under N deficiency, the level of 36:5-PG increased in root hairs (Fig. 4A; Supplemental Table 2). With sufficient N, the level of 36:6-PE was higher whereas that of 36:4-PI was lower than that of root hairs with deficient N (Fig. 4B; Supplemental Table 2). The levels of lysophospholipids LPG, LPC, and LPE were comparable between N-sufficient and -deficient conditions, with significant decreases occurring to 16:1-LPG, and increased to 16:0-LPC and 18:2-LPC under N deficiency (Fig. 4D; Supplemental Table 2).

By comparison, the overall levels of major membrane lipids in stripped roots were not significantly different between N-sufficient and -deficient conditions, except that the level of LPC increased in N-sufficient conditions. However, differences occurred at the molecular species level in stripped roots. With sufficient N, the levels of 34:1-MGDG, 36:1-MGDG, 38:5-MGDG and 36:3-PG increased in stripped roots (Fig. 4A; Supplemental Table 2). Some PC, PE, PS, PI and PA species displayed significant differences under N deficiency. The levels of 32:0-PC and 34:3-PC were higher than that under N deficiency whereas those of 32:3-PE and 36:5-PE were lower. The levels of 32:0-PI and 34:4-PI increased but those of 36:6-PI decreased under N deficiency (Fig. 4B; Supplemental Table 2). Most PA and PS species were unchanged, but the levels of 34:2-PS, 36:6-PS, 34:6-PA and 34:4-PA were higher under N deficiency while that of 42:1-PS was lower (Fig. 4C; Supplemental Table 2). The levels of lysophospholipids were comparable between N-sufficient and -deficient conditions, with significant increases occurring to 16:0-LPG and 18:2-LPE, and a decrease to 18:3-LPC under N deficiency (Fig. 4D; Supplemental Table 2).

### Glycerolipid Changes in Root Hairs and Stripped Roots Affected by P_i_ Supplement

**Figure 5:**
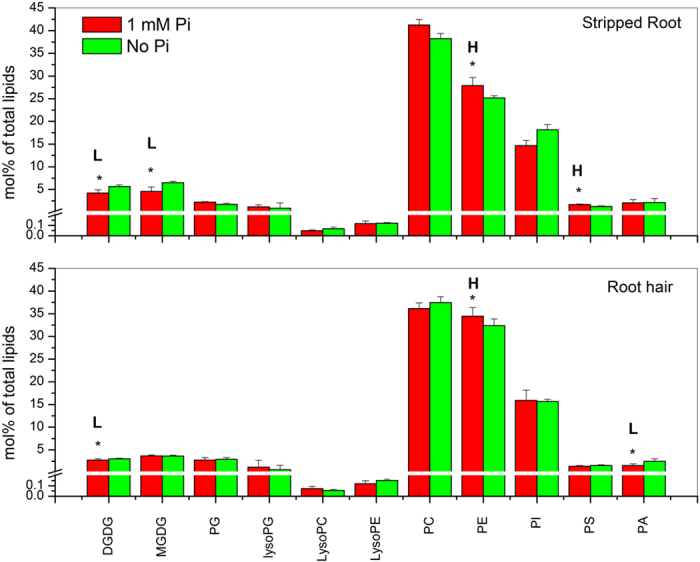
Glycerolipid classes in soybean root hairs and stripped roots with and without P_i_ supply. Lipids were extracted from stripped roots and root hairs collected from 7-day-old seedlings grown on modified Murashige and Skoog agar medium with 1 mM P_i_ or no P_i_. Glycerolipid amounts are expressed as normalized mass spectral signal/total normalized glycerolipid mass spectral signal (to produce percentage of normalized MS signal, mol% of total lipids). The values are the mean ± SD (n = 10). The data of soybean stripped roots and root hairs were compared via *t* test and the P < 0.05 is indicated by *, indicating a significant difference. The value for P_i_-sufficient seedlings is higher (represented as H) or lower (represented as L) than the value for P_i_-deficient seedlings.

To explore the effect of P_i_ availability on membrane glycerolipid changes in root hairs, we germinated soybean seeds in agar media 0.8% (w/v) with or without added P_i_ for seven days. At this stage, more root hairs were observed under P_i_-limited than P_i_-sufficient conditions. Stripped roots and root hairs were collected and lipids analyzed are presented in Supplemental Table 3. When soybean seedlings were grown without supplied P_i_, the level of total phospholipids decreased in stripped roots, whereas that of galactolipids increased. The ratio of galactolipids to phospholipids in stripped roots increased 1.4 fold. The increase came primarily from increases in DGDG and MGDG whereas the levels of PE and PS decreased 10% and 25%, respectively (Fig. 5; Supplemental Table 3). By comparison, the galactolipid to phospholipid ratio increase in root hairs was smaller than that in stripped roots. The increase in root hairs came primarily from an increase in the galactolipid DGDG (11%) and a decline in the phospholipid PE (6%). However, the content of PA increased by 60% under P_i_ deprivation (Fig. 5; Supplemental Table 3).

**Figure 6:**
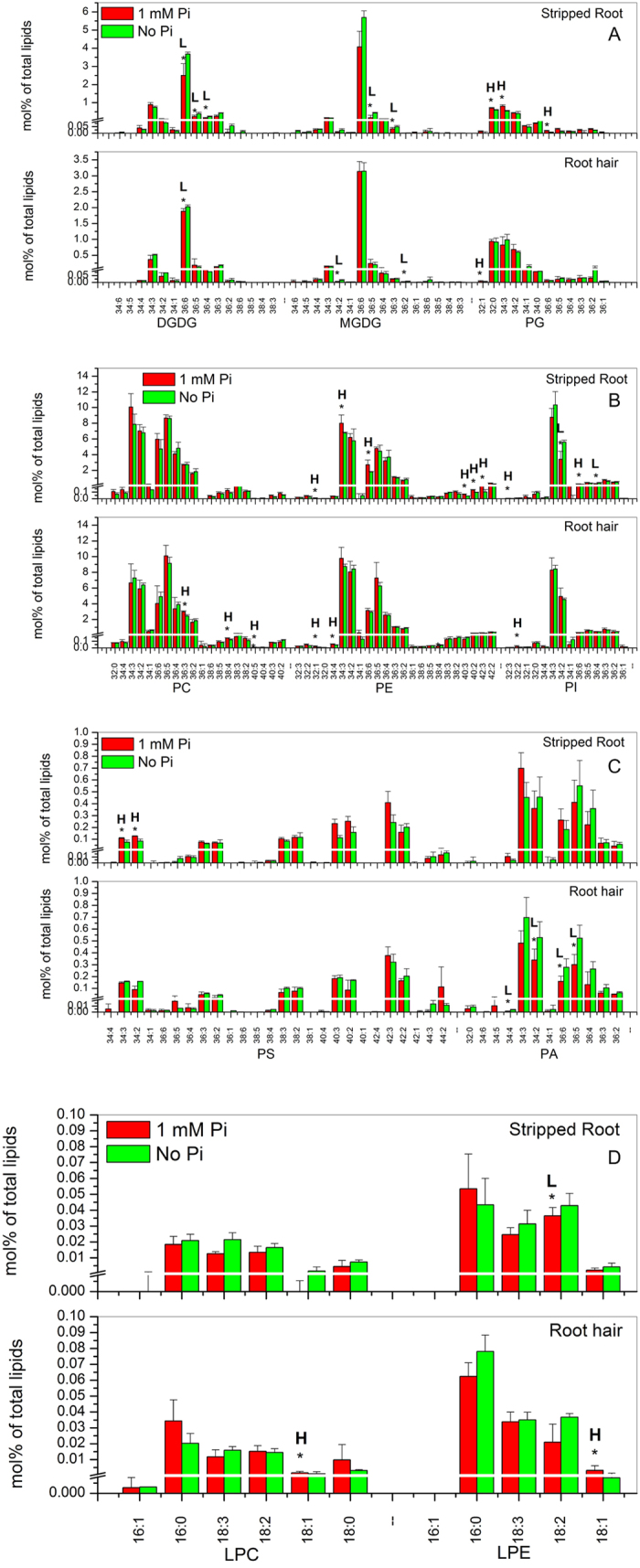
Glycerolipid molecular species in soybean root hairs and stripped roots with and without supplying P_i_. Lipids were extracted from stripped roots and root hairs collected from 7-day-old seedlings grown on modified Murashige and Skoog agar medium with 1 mM P_i_ or no P_i_. Glycerolipid amounts are expressed as normalized mass spectral signal/total normalized glycerolipid mass spectral signal (to produce percentage of normalized MS signal, mol% of total lipids). The values are the mean ± SD (n = 10). The data of soybean stripped roots and root hairs were compared via *t* test and the P < 0.05 is indicated by *, indicating a significant difference. The value for P_i_-sufficient seedlings is higher (represented as H) or lower (represented as L) than the value for P_i_-deficient seedlings.

In stripped roots, under P_i_ deprivation, the levels of some DGDG and MGDG species, such as 36:6-DGDG, 36:5-DGDG, 36:4-DGDG, 36:5-MGDG and 36:3-MGDG, increased, whereas those of 32:0-PG, 34:3-PG, and 36:6-PG decreased, compared to the P_i_-sufficient condition (Fig. 6A; Supplemental Table 3). The levels of 32:1-PE, 34:3-PE, 36:6-PE, 40:3-PE, 40:2-PE and 42:3-PE also increased under P_i_ deficiency. The levels of 34:2-PI and 36:4-PI increased but that of 32:3-PI and 36:6-PI decreased under P_i_ deficiency (Fig. 6B; Supplemental Table 3). Most PA and PS species were unchanged except 34:3-PS and 34:2-PS that were lower under P_i_ deficiency (Fig. 6C; Supplemental Table 3). The levels of lysophospholipids were comparable between P_i_-sufficient and -deficient conditions, with a significant decrease in 16:0-LPG, and an increase in 18:2-LPE under P_i_ deficiency (Fig. 6D; Supplemental Table 3).

In root hairs, P_i_ deficiency induced a decrease in the level of galactolipid species, such as 36:6-DGDG, 34:2-MGDG, and 36:2-MGDG (Fig. 6A; Supplemental Table 3). However, the levels of phospholipid species, such as 36:3-PC, 38:4-PC, 40:5-PC, 32:1-PE, 34:4-PE and 32:2-PI, were higher under P_i_ deficiency than those under P_i_-sufficient conditions (Fig. 6B; Supplemental Table 3). No significant difference in PS species occurred between P_i_-sufficient and P_i_-deficient conditions. The levels of 34:4-PA, 34:2-PA, 36:6-PA and 36:5-PA were lower than the levels under P_i_ deficiency (Fig. 6C; Supplemental Table 3). The levels of lysophospholipids were comparable between P_i_-sufficient and -deficient conditions, with significant decreases occurring to 18:1-LPC and 18:1-LPE under P_i_ deficiency (Fig. 6D; Supplemental Table 3).

## Discussion

The study was initiated to analyze the glycerolipidome of the single cell type root hairs and to compare the glycerolipidomic composition between root hairs and stripped roots. Root hairs are a single, terminally differentiated plant cell type and they are the extension of root epidermal cells. Root hairs and stripped roots exhibit distinctive differences in lipid species. The ratio of phospholipids to galactolipids was 1.5 fold higher in root hairs than in stripped roots. PG and PE are more abundant in root hairs than in stripped roots. On the other hand, MGDG, DGDG, PC and PS were more abundant in stripped roots compared with root hairs. MGDG and DGDG are located in plastids and the higher level of galactolipids may result from the presence of a higher level of plastids in stripped roots than in root hairs. In addition, compared with stripped roots, root hairs have a lower level of lipids with long chain fatty acid species, such as 36:6-DGDG, 36:4-DGDG, 36:3-DGDG, 36:6-MGDG, 36:4-MGDG, 36:3-PE, 36:2-PS, 44:3-PS and 36:6-PA. The synthesis of very long chained fatty acids requires KASIII, and this decrease might mean a lower activity of fatty acid elongation in root hairs. In addition, membranes with the shorter fatty acid chains in root hairs are expected to be more fluid than those of stripped roots. The increased fluidity may help support the rapid root hair growth.

In addition, this study analyzed how the membrane glycerolipidome of root hairs responds to N or P_i_ availability since root hairs play an important role in nutrient absorption. The availability of N affects greatly carbohydrate and protein metabolism, but its effect on lipids remains poorly understood. Glycerophospholipids, such as PC, PE, and PS, contain N in the head group. The level of galactolipids decreased in N-deprived Arabidopsis seedlings^5^. The decrease in galactolipids occurred also with soybean plants whereas the content of total phospholipids remained relatively constant in N-sufficient and -deficient growth conditions in soybean plants^8^. In contrast, in soybean root hairs without N supply, the level of the N-containing lipids PE and PS decreased whereas the level of non-N-containing lipids DGDG, MGDG, and PA increased compared to N-sufficient conditions. By comparison, the overall level of major membrane lipids in stripped roots was not different between N-sufficient and -deficient conditions. The difference between root hairs and stripped roots suggests that membrane glycerolipidomes in root hairs are more responsive to N availability than are the main body of roots. This result is in agreement with a recent transcriptome analysis of wheat under drought, suggesting that root hairs play a role as sensors of environmental conditions^19^.

Membrane phospholipids contain approximately one third of organic P_i_ in plants. In response to P_i_ starvation, the level of cellular phospholipids decreases, and the decrease is partially compensated for by an increase in non-phosphorus containing lipids, such as DGDG, to maintain membrane integrity^22^,^23^,^24^,^25^. Under P_i_ deficiency, the ratio of galactolipids to phospholipids in stripped roots increased 1.4 fold, and the change in ratio is consistent with general plant response to P_i_ deprivation. However, the change in specific lipid classes differed. While PC in Arabidopsis is the class of phospholipids that decreased the most^26^,^27^, the level of PE, the major lipid, decreased in soybean stripped roots and in root hairs. A noted difference between root hairs and stripped roots is the PA level as affected by P_i_ deprivation. The level of PA in root hairs displayed an increase under P_i_ deprivation, but no such change occurred in stripped roots. PA is a central intermediate in glycerolipid metabolism and also a key mediator in plant response to stress, including P_i_ availability. In Arabidopsis under moderate P_i_ deprivation, the PA level increased and knockout of PLDζs abolished the difference in PA, indicating PLDζs are responsible for the elevated PA production^6^. PLDζs promote root and root hair growth in response to P_i_ deprivation^26^,^27^. Soybean has three PX/PH-PLDζs, and two of them are more closely related to AtPLDζ1 and one closely resembles AtPLDζ2^28^. The higher level of PA could mean that PLDζs are more responsive to P_i_ availability in root hairs than in the rest of the roots.

The increase in PA in both P_i_- and N-deficient conditions could impact root growth and proliferation via its impact on vesicular trafficking and cytoskeletal reorganization. In a proteomic analysis of soybean root hairs, a PLDα was identified to respond to *B. japonicum* inoculation^14^. PLD and PA have been implicated in nodulation^29^ through its functions in cell signaling and cytoskeletal reorganization in plant cells^30^, which may play a role in root-hair deformation induced by compatible rhizobia^29^. Under salt stress, PA targets both MAPK and MAP65-1 to regulate microtubule polymerization and bundling^29^,^31^. PA may mediate the formation of membrane lipid-cytoskeleton interfaces to coordinately regulate subcellular dynamics. PA in mammalian systems affects vesicle trafficking-related processes involved in exocytosis, endocytosis, membrane fusion, and vesicle budding^32^,^33^. In Arabidopsis, the ADP-ribosylation factor (ARF) GTPase-activating protein 7 (AGD7) regulates ARF1 that is involved in vesicle trafficking and fusion in a PA-dependent manner^34^. In addition, PA binds to the protein phosphatase PP2AA1 to regulate the trafficking and polar localization of PIN1 in auxin transport^35^. Furthermore, the negatively charged and cone-shaped PAs can affect vesicle formation and membrane fission and fusion^36^. Under P_i_ and N deficiency, root hair length and number are increased, and the increase in PA may promote membrane trafficking that is required for fast growing in root hairs.

## Materials and Methods

### Plant Growth and N Treatments

Soybean seeds (*Glycine max* cv Jack) were sterilized by soaking seeds twice in 20% bleach for 10 min each. Seeds were then rinsed five times in sterile water, neutralized for 10 min in 0.1 N HCl, and rinsed five more times in sterile water. Sterilized seeds were germinated in a dark growth chamber (25 °C, 80% humidity) for 4 d on nitrogen-free B&D agar medium^21^ solidified with 0.8% (w/v) agarose in 18 cm × 18 cm Petri dishes sealed with Parafilm. Then 20 mM nitrogen (10 mM NH_4_NO_3_) was supplied to 4-day-old seedlings using a sprayer and control seedlings were sprayed with water. After 12 hours, stripped roots and root hairs were collected from 4-day-old seedlings treated with or without 20 mM N for lipid profiling. For each treatment, five biological replicates were included.

### Plant Growth and P_i_ Treatments

Sterilized seeds were germinated in a dark growth chamber (25 °C, 80% humidity) for 7 d on normal modified Murashige and Skoog agar medium either with or without P_i_. The modified medium contained 1.25 mM KNO_3_, 1.5 mM Ca(NO_3_)_2_, 0.75 mM MgSO_4_, 1 mM KH_2_PO4, 75 mM FeEDTA, 50 mM H_3_BO_3_, 10 mM MnCl, 2 mM ZnSO_4_, 1.5 mM CuSO_4_, and 0.075 mM (NH_4_)_6_Mo_7_O_24_. The P_i_-depleted medium contained 1 mM KCl instead of KH_2_PO_4_, solidified with 0.8% (w/v) agarose in 18 cm × 18 cm Petri dishes sealed with Parafilm. Stripped roots and root hairs were collected from 7-day-old seedlings grown on the above media for lipid profiling. For each treatment, five biological replicates were included.

### Root Hairs and Stripped Root Isolation

Root hairs were isolated according to the procedure described previously^14^,^17^. Briefly, soybean roots (about 200 per experiment) were collected by cutting and allowing the roots to fall directly into liquid nitrogen. The roots were gently stirred for 20 min to break off root hairs from roots. The liquid nitrogen slurry was filtered through a wire mesh to separate root hairs from stripped roots. Root hairs and stripped roots were stored at −80 °C until lipid extraction.

### Lipid Extraction and Profiling

Lipids were extracted from stripped roots and root hairs of soybean as described in a previous report with some modifications^37^. Briefly, stripped roots and root hairs were immersed immediately into 3 mL 75 °C (preheated) isopropanol with 0.01% butylated hydroxytoluene for 15 min to inhibit lipolytic activities. Chloroform (CHCl_3_; 1.5 mL) and H_2_O (0.6 mL) were individually added to samples, which were placed on a shaker for 1 h. After each extracting solvent was transferred to a new tube, samples were re-extracted with CHCl_3_:CH_3_OH (2:1) at least 4 times, with 30 min of agitation each time. Extracts for each sample were combined and washed with 1 M KCl followed by H_2_O (1 mL). Solvent was evaporated under N_2_ to concentrate lipids, and each lipid sample finally was dissolved in 1 mL CHCl_3_. Each tube with plant tissue residue was dried at 105 °C overnight and weighed with a precision balance to determine the total tube weight and plant tissue weight. Because the root hair is too small to transfer from the tubes for weighing without a loss, dry weight was obtained by the total weight of tubes and plant tissue minus the weight of the empty tubes.

Lipid samples were analyzed using an electrospray ionization triple quadrupole mass spectrometer (API 4000; Applied Biosystems, Foster City, CA) as described previously^33^. The molecular species of phospholipids and galactolipids were quantified in comparison to internal standards. The internal standards for galactolipids were 2.01 nmol 16:0–18:0-MGDG, 0.39 nmol di18:0-MGDG, 0.49 nmol 16:0–18:0-DGDG, and 0.71 nmol di18:0-DGDG, purchased from Matreya, Inc. (State College, PA). The internal standards for phospholipids were 0.66 nmol di14:0-PC, 0.66 nmol di24:1-PC, 0.66 nmol 13:0-LPC, 0.66 nmol 19:0-LPC, 0.36 nmol di14:0-PE, 0.36 nmol di24:1-PE, 0.36 nmol 14:0-LPE, 0.36 nmol 18:0-LPE, 0.36 nmol di14:0-PG, 0.36 nmol di24:1-PG, 0.36 nmol 14:0-LPG, 0.36 nmol 18:0-LPG, 0.36 nmol di14:0-PA, 0.36 nmol di20:0 (phytanoyl)-PA, 0.24 nmol di14:0-PS, 0.24 nmol di20:0 (phytanoyl)-PS, 0.20 nmol 16:0–18:0-PI and 0.16 nmol di18:0-PI. All phospholipid standards were obtained from Avanti Polar Lipids, Inc. (Alabaster, AL), except for di24:1-PE and di24:1-PG, which were prepared by transphosphatidylation of di24:1-PC. The quantity of each lipid was determined as a normalized mass spectral signal (i.e. normalized to the two internal standards of that class), as described earlier^37^,^38^. The normalized signal was divided by total normalized signal (to produce percentage of normalized MS signal). This approach allows comparison of quantities of lipid species and classes among samples. Five biological replications of each treatment were processed for each analysis experiment and the experiment was repeated twice.

### Statistical Analyses

The pairwise comparison of the lipids data of soybean stripped root and root hair, the lipids data of nitrogen/phosphate sufficient and deficient conditions (stripped root and root hair respectively) were via *t* test to determine the statistical significance.

## Additional Information

**How to cite this article**: Wei, F. *et al*. Membrane glycerolipidome of soybean root hairs and its response to nitrogen and phosphate availability. *Sci. Rep*. **6**, 36172; doi: 10.1038/srep36172 (2016).

**Publisher’s note:** Springer Nature remains neutral with regard to jurisdictional claims in published maps and institutional affiliations.

## Supplementary Material

Supplementary Information
