# Supplementary material for: Membrane glycerolipidome of soybean root hairs and its response to nitrogen and phosphate availability
Source: Sci Rep. 2016 Nov 4;6:36172. doi: 10.1038/srep36172 (PMC5095881; doi:10.1038/srep36172)
Supplement: Supplementary Information [file srep36172-s1.doc]

**Supplementary data for:**

**Membrane glycerolipidome of soybean root hairs and its response to nitrogen and phosphate availability**

Fang Wei, Brian Fanella, Liang Guo and Xuemin Wang

**
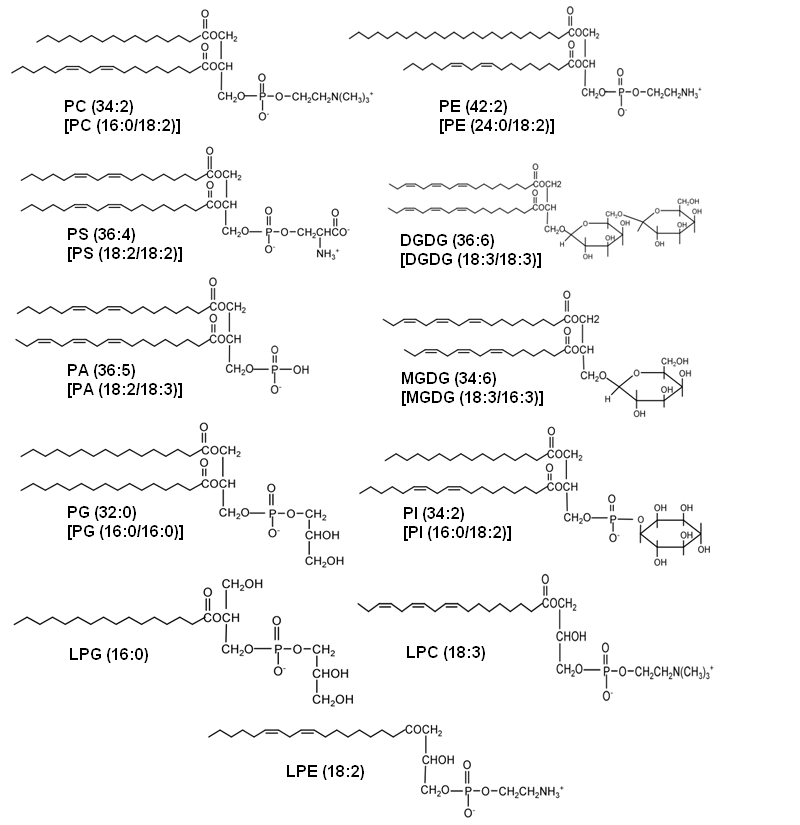
**

Supplemental Fig. 1 Structures representing each class of lipids. The total acyl carbons: total double bonds have been provided for each compound and the corresponding lipid class. This figure was modified from a figure in reference 3.

**Supplementary Table 1:** **Amounts of polar lipid molecular species of soybean stripped root and root hair.** Stripped Roots and root hairs collected from 7-day-old seedlings grown on normal Murashige and Skoog agar medium conditions were used to analyze the lipids. Five biological replications were maintained for each experiment and the experiment was repeated twice. The lipids were analyzed by ESI-MS/MS and the intensities in each spectrum were normalized to those two internal standards of the same class; a signal of 1 is the same amount of intensity as 1 nmol of the standards. The data represents the average of 10 replicates. The data of soybean stripped root and root hair were compared via *t* test and the P values are reported. The P value <0.05 is presented in bold. The reduction in lipid species content of stripped root is denoted as minus (-) sign.

| Lipid species (class and total acyl carbons: total carbon-carbon double bonds) | Compound Number | Polar lipid molecular species composition (mol %)  (polar lipid molecular species as % (mol/mol) total polar lipid molecular species) | | | |
| --- | --- | --- | --- | --- | --- |
|  |  | Stripped root | Root hair | P value | Difference between stripped root and root hair |
| PC 32:0 | 1 | 0.108 | 0.076 | 0.496 | 0.032 |
| PC 34:4 | 2 | 0.136 | 0.095 | 0.430 | 0.041 |
| PC 34:3 | 3 | 10.060 | 6.662 | 0.329 | 3.398 |
| PC 34:2 | 4 | 7.035 | 5.897 | 0.391 | 1.138 |
| PC 34:1 | 5 | 0.222 | 0.481 | 0.103 | -0.259 |
| PC 36:6 | 6 | 5.964 | 4.028 | 0.205 | 1.936 |
| PC 36:5 | 7 | 8.665 | 10.104 | 0.093 | -1.439 |
| PC 36:4 | 8 | 4.087 | 3.342 | 0.124 | 0.745 |
| PC 36:3 | 9 | 2.708 | 2.973 | **0.017** | -0.265 |
| PC 36:2 | 10 | 1.589 | 1.650 | 0.497 | -0.061 |
| PC 36:1 | 11 | 0 | 0.038 | 0.211 | -0.038 |
| PC 38:6 | 12 | 0.043 | 0.036 | 0.305 | 0.007 |
| PC 38:5 | 13 | 0.087 | 0.092 | 0.258 | -0.005 |
| PC 38:4 | 14 | 0.125 | 0.146 | 0.109 | -0.021 |
| PC 38:3 | 15 | 0.186 | 0.170 | 0.202 | 0.016 |
| PC 38:2 | 16 | 0.109 | 0.144 | 0.117 | -0.035 |
| PC 40:5 | 17 | 0.006 | 0.010 | **0.013** | -0.004 |
| PC 40:4 | 18 | 0.009 | 0.010 | 0.147 | -0.001 |
| PC 40:3 | 19 | 0.049 | 0.081 | **0.021** | -0.032 |
| PC 40:2 | 20 | 0.081 | 0.090 | 0.223 | -0.009 |
| **Total PC** |  | 41.266 | 36.125 | **0.038** | 5.141 |
| PE 32:3 | 21 | 0.022 | 0.026 | 0.291 | -0.004 |
| PE 32:2 | 22 | 0.043 | 0.047 | 0.166 | -0.004 |
| PE 32:1 | 23 | 0.011 | 0.019 | 0.111 | -0.008 |
| PE 32:0 | 24 | 0.005 | 0.005 | 0.365 | 0 |
| PE 34:4 | 25 | 0.035 | 0.053 | 0.091 | -0.018 |
| PE 34:3 | 26 | 8.018 | 9.781 | 0.109 | -1.763 |
| PE 34:2 | 27 | 6.198 | 8.068 | **0.049** | -1.87 |
| PE 34:1 | 28 | 0 | 0.295 | 0.160 | -0.295 |
| PE 36:6 | 29 | 2.727 | 3.145 | 0.129 | -0.418 |
| PE 36:5 | 30 | 4.790 | 7.279 | 0.170 | -2.489 |
| PE 36:4 | 31 | 3.219 | 2.554 | 0.121 | 0.665 |
| PE 36:3 | 32 | 1.130 | 1.036 | **0.035** | 0.094 |
| PE 36:2 | 33 | 0.694 | 0.787 | 0.454 | -0.093 |
| PE 36:1 | 34 | 0.033 | 0.016 | 0.416 | 0.017 |
| PE 38:6 | 35 | 0.016 | 0.016 | 0.379 | 0 |
| PE 38:5 | 36 | 0.031 | 0.030 | 0.314 | 0.001 |
| PE 38:4 | 37 | 0.026 | 0.026 | 0.074 | 0 |
| PE 38:3 | 38 | 0.077 | 0.124 | **0.049** | -0.047 |
| PE 38:2 | 39 | 0.106 | 0.144 | 0.181 | -0.038 |
| PE 40:3 | 40 | 0.067 | 0.130 | **0.045** | -0.063 |
| PE 40:2 | 41 | 0.130 | 0.266 | **0.008** | -0.136 |
| PE 42:3 | 42 | 0.190 | 0.285 | **0.013** | -0.095 |
| PE 42:2 | 43 | 0.352 | 0.332 | 0.275 | 0.02 |
| **Total PE** |  | 27.918 | 34.466 | **0.043** | -6.548 |
| PS 34:4 | 44 | 0 | 0.005 | 0.211 | -0.005 |
| PS 34:3 | 45 | 0.110 | 0.147 | **0.003** | -0.037 |
| PS 34:2 | 46 | 0.126 | 0.090 | 0.161 | 0.036 |
| PS 34:1 | 47 | 0 | 0.003 | 0.151 | -0.003 |
| PS 36:6 | 48 | 0 | 0.002 | 0.211 | -0.002 |
| PS 36:5 | 49 | 0.001 | 0.018 | 0.198 | -0.017 |
| PS 36:4 | 50 | 0.011 | 0.007 | 0.069 | 0.004 |
| PS 36:3 | 51 | 0.078 | 0.048 | 0.169 | 0.03 |
| PS 36:2 | 52 | 0.071 | 0.022 | **0.048** | 0.049 |
| PS 36:1 | 53 | 0 | 0 | 0.195 | 0 |
| PS 38:6 | 54 | 0.001 | 0 | 0.402 | 0.001 |
| PS 38:5 | 55 | 0 | 0 | 0.211 | 0 |
| PS 38:4 | 56 | 0.003 | 0.002 | 0.123 | 0.001 |
| PS 38:3 | 57 | 0.105 | 0.066 | 0.128 | 0.039 |
| PS 38:2 | 58 | 0.118 | 0.077 | 0.094 | 0.041 |
| PS 38:1 | 59 | 0 | 0 | 0.168 | 0 |
| PS 40:4 | 60 | 0 | 0.001 | 0.298 | -0.001 |
| PS 40:3 | 61 | 0.233 | 0.182 | 0.470 | 0.051 |
| PS 40:2 | 62 | 0.252 | 0.086 | 0.165 | 0.166 |
| PS 40:1 | 63 | 0 | 0 | 0.318 | 0 |
| PS 42:4 | 64 | 0 | 0 | 0.211 | 0 |
| PS 42:3 | 65 | 0.409 | 0.377 | 0.292 | 0.032 |
| PS 42:2 | 66 | 0.159 | 0.164 | 0.245 | -0.005 |
| PS 42:1 | 67 | 0 | 0 | 0.153 | 0 |
| PS 44:3 | 68 | 0.008 | 0.001 | **0.043** | 0.007 |
| PS 44:2 | 69 | 0.014 | 0.112 | 0.225 | -0.098 |
| **Total PS** |  | 1.697 | 1.412 | **0.045** | 0.285 |
| DGDG 34:6 | 70 | 0.002 | 0 | 0.290 | 0.002 |
| DGDG 34:5 | 71 | 0 | 0 | 0.198 | 0 |
| DGDG 34:4 | 72 | 0.040 | 0.013 | 0.353 | 0.0274 |
| DGDG 34:3 | 73 | 0.895 | 0.368 | **0.038** | 0.527 |
| DGDG 34:2 | 74 | 0.098 | 0.046 | 0.095 | 0.052 |
| DGDG 34:1 | 75 | 0.027 | 0.013 | 0.398 | 0.014 |
| DGDG 36:6 | 76 | 2.497 | 1.882 | **0.044** | 0.615 |
| DGDG 36:5 | 77 | 0.239 | 0.190 | 0.484 | 0.049 |
| DGDG 36:4 | 78 | 0.144 | 0.090 | **0.035** | 0.054 |
| DGDG 36:3 | 79 | 0.253 | 0.128 | **0.049** | 0.125 |
| DGDG 36:2 | 80 | 0.007 | 0.009 | 0.295 | -0.002 |
| DGDG 38:6 | 81 | 0.001 | 0.005 | 0.186 | -0.004 |
| DGDG 38:5 | 82 | 0 | 0 | 0.312 | 0 |
| DGDG 38:4 | 83 | 0 | 0 | 0.252 | 0 |
| DGDG 38:3 | 84 | 0 | 0 | 0.224 | 0 |
| **Total DGDG** |  | 4.204 | 2.743 | **0.026** | 1.461 |
| PA 32:0 | 85 | 0 | 0.006 | 0.382 | -0.006 |
| PA 34:6 | 86 | 0 | 0 | 0.313 | 0 |
| PA 34:5 | 87 | 0 | 0.010 | 0.205 | -0.01 |
| PA 34:4 | 88 | 0.011 | 0.001 | 0.256 | 0.01 |
| PA 34:3 | 89 | 0.698 | 0.482 | **0.042** | 0.216 |
| PA 34:2 | 90 | 0.360 | 0.339 | 0.171 | 0.021 |
| PA 34:1 | 91 | 0 | 0.002 | 0.298 | -0.002 |
| PA 36:6 | 92 | 0.263 | 0.160 | **0.039** | 0.103 |
| PA 36:5 | 93 | 0.412 | 0.301 | 0.112 | 0.111 |
| PA 36:4 | 94 | 0.222 | 0.133 | 0.114 | 0.089 |
| PA 36:3 | 95 | 0.067 | 0.061 | 0.222 | 0.006 |
| PA 36:2 | 96 | 0.045 | 0.050 | 0.249 | -0.005 |
| **Total PA** |  | 2.078 | 1.544 | 0.117 | 0.534 |
| MGDG 34:6 | 97 | 0.002 | 0.008 | 0.170 | -0.006 |
| MGDG 34:5 | 98 | 0.005 | 0.007 | 0.237 | -0.002 |
| MGDG 34:4 | 99 | 0.026 | 0.022 | 0.172 | 0.004 |
| MGDG 34:3 | 100 | 0.144 | 0.144 | 0.223 | 0 |
| MGDG 34:2 | 101 | 0.007 | 0.006 | 0.148 | 0.001 |
| MGDG 34:1 | 102 | 0.005 | 0.002 | 0.492 | 0.003 |
| MGDG 36:6 | 103 | 4.074 | 3.136 | **0.046** | 0.938 |
| MGDG 36:5 | 104 | 0.170 | 0.240 | 0.109 | -0.07 |
| MGDG 36:4 | 105 | 0.100 | 0.070 | **0.037** | 0.03 |
| MGDG 36:3 | 106 | 0.033 | 0.025 | 0.473 | 0.008 |
| MGDG 36:2 | 107 | 0 | 0.003 | 0.446 | -0.003 |
| MGDG 36:1 | 108 | 0.007 | 0.001 | 0.087 | 0.006 |
| MGDG 38:6 | 109 | 0.013 | 0.006 | 0.098 | 0.007 |
| MGDG 38:5 | 110 | 0.002 | 0.001 | 0.258 | 0.001 |
| MGDG 38:4 | 111 | 0.004 | 0 | 0.211 | 0.004 |
| MGDG 38:3 | 112 | 0 | 0.002 | 0.227 | -0.002 |
| **Total MGDG** |  | 4.592 | 3.672 | **0.043** | 0.92 |
| PG 32:1 | 113 | 0.013 | 0.007 | 0.053 | 0.006 |
| PG 32:0 | 114 | 0.703 | 0.938 | **0.014** | -0.235 |
| PG 34:3 | 115 | 0.807 | 0.825 | 0.331 | -0.018 |
| PG 34:2 | 116 | 0.432 | 0.683 | 0.138 | -0.251 |
| PG 34:1 | 117 | 0.055 | 0.090 | 0.432 | -0.035 |
| PG 34:0 | 118 | 0.074 | 0.080 | 0.169 | -0.006 |
| PG 36:6 | 119 | 0.019 | 0.015 | 0.232 | 0.004 |
| PG 36:5 | 120 | 0.032 | 0.023 | 0.102 | 0.009 |
| PG 36:4 | 121 | 0.014 | 0.022 | 0.144 | -0.008 |
| PG 36:3 | 122 | 0.024 | 0.033 | 0.328 | -0.009 |
| PG 36:2 | 123 | 0.031 | 0.032 | 0.356 | -0.001 |
| PG 36:1 | 124 | 0.007 | 0.005 | 0.057 | 0.002 |
| **Total PG** |  | 2.214 | 2.756 | **0.030** | -0.542 |
| PI 32:3 | 125 | 0.006 | 0.005 | **0.043** | 0.001 |
| PI 32:2 | 126 | 0.004 | 0.021 | 0.158 | -0.017 |
| PI 32:1 | 127 | 0.036 | 0.008 | 0.201 | 0.028 |
| PI 32:0 | 128 | 0.068 | 0.070 | 0.231 | -0.002 |
| PI 34:4 | 129 | 0.014 | 0.021 | 0.201 | -0.007 |
| PI 34:3 | 130 | 8.768 | 8.287 | 0.312 | 0.481 |
| PI 34:2 | 131 | 3.428 | 4.933 | 0.174 | -1.505 |
| PI 34:1 | 132 | 0.181 | 0.051 | 0.414 | 0.13 |
| PI 36:6 | 133 | 0.261 | 0.315 | 0.147 | -0.054 |
| PI 36:5 | 134 | 0.395 | 0.5460 | **0.011** | -0.151 |
| PI 36:4 | 135 | 0.306 | 0.4282 | **0.042** | -0.122 |
| PI 36:3 | 136 | 0.761 | 0.752 | 0.278 | 0.009 |
| PI 36:2 | 137 | 0.443 | 0.428 | 0.495 | 0.015 |
| PI 36:1 | 138 | 0 | 0.019 | 0.211 | -0.019 |
| **Total PI** |  | 14.673 | 15.883 | 0.328 | -1.21 |
| LPG 16:1 | 139 | 0 | 0.029 | 0.211 | -0.029 |
| LPG 16:0 | 140 | 0.388 | 0.533 | 0.062 | -0.145 |
| LPG 18:3 | 141 | 0.185 | 0.552 | 0.173 | -0.367 |
| LPG 18:2 | 142 | 0 | 0 | 0.167 | 0 |
| LPG 18:1 | 143 | 0.619 | 0.091 | **0.010** | 0.528 |
| **Total LPG** |  | 1.192 | 1.204 | 0.057 | -0.012 |
| LPC(16:1) | 144 | 0 | 0 | 0.138 | -0.0003 |
| LPC(16:0) | 145 | 0.018 | 0.034 | 0.199 | -0.016 |
| LPC(18:3) | 146 | 0.013 | 0.012 | 0.400 | 0.001 |
| LPC(18:2) | 147 | 0.013 | 0.015 | 0.495 | -0.002 |
| LPC(18:1) | 148 | 0 | 0.002 | **0.011** | -0.002 |
| LPC(18:0) | 149 | 0.005 | 0.010 | 0.162 | -0.005 |
| **TOTAL LPC** |  | 0.049 | 0.073 | 0.139 | -0.024 |
| LPE(16:1) | 150 | 0 | 0 | 0.156 | 0 |
| LPE(16:0) | 151 | 0.053 | 0.062 | 0.469 | -0.009 |
| LPE(18:3) | 152 | 0.025 | 0.034 | 0.202 | -0.009 |
| LPE(18:2) | 153 | 0.036 | 0.021 | 0.266 | 0.015 |
| LPE(18:1) | 154 | 0.002 | 0.003 | 0.071 | -0.001 |
| **TOTAL LPE** |  | 0.117 | 0.120 | 0.495 | -0.003 |

**Supplementary Table 2:** **Amounts of polar lipid molecular species as affected by nitrogen starvation.** 4-day-old seedlings grown on nitrogen-free B&D agar medium were treated with 20 mM nitrogen (nitrogen sufficient conditions) and sterile water (nitrogen deficient conditions). After 12 hours treatment, stripped roots and root hairs were collected for lipid profiling. Five biological replications were maintained for each experiment and the experiment was repeated twice. The lipids were analyzed by ESI-MS/MS and the intensities in each spectrum were normalized to those two internal standards of the same class; a signal of 1 is the same amount of intensity as 1 nmol of the standards. The data represents the average of 10 replicates. The data of nitrogen sufficient and deficient conditions were compared via *t* test and the P values are reported. The P value <0.05 is presented in bold.

| **Lipid species (class and total acyl carbons: total carbon-carbon double bonds)** | **Compound Number** | Polar lipid molecular species composition (mol %)  (polar lipid molecular species as % (mol/mol) total polar lipid molecular species) | | | | | | | |
| --- | --- | --- | --- | --- | --- | --- | --- | --- | --- |
|  |  | **Stripped Root** | | | | **Root hair** | | | |
|  |  | **N**  **20 mM** | **N**  **0 mM** | **P value** | **Difference between N 20 mM and N 0 mM** | **N**  **20 mM** | **N**  **0 mM** | **P value** | **Difference between N 20 mM and N 0 mM** |
| PC 32:0 | 1 | 0.127 | 0.105 | **0.012** | 0.022 | 0.085 | 0.072 | 0.397 | 0.013 |
| PC 34:4 | 2 | 0.093 | 0.111 | 0.129 | -0.018 | 0.114 | 0.12 | 0.094 | -0.006 |
| PC 34:3 | 3 | 8.429 | 8.011 | **0.045** | 0.418 | 9.245 | 9.866 | 0.127 | -0.621 |
| PC 34:2 | 4 | 6.231 | 6.089 | 0.132 | 0.142 | 6.734 | 6.708 | 0.16 | 0.026 |
| PC 34:1 | 5 | 0.433 | 0.467 | 0.45 | -0.034 | 0.373 | 0.288 | 0.343 | 0.085 |
| PC 36:6 | 6 | 5.899 | 5.714 | 0.216 | 0.185 | 5.786 | 5.862 | 0.284 | -0.076 |
| PC 36:5 | 7 | 8.005 | 7.982 | 0.344 | 0.023 | 7.798 | 7.74 | 0.158 | 0.058 |
| PC 36:4 | 8 | 4.021 | 4.283 | 0.201 | -0.262 | 4.006 | 3.774 | 0.162 | 0.232 |
| PC 36:3 | 9 | 2.749 | 2.808 | 0.443 | -0.059 | 2.842 | 2.754 | 0.499 | 0.088 |
| PC 36:2 | 10 | 1.575 | 1.611 | 0.336 | -0.036 | 1.579 | 1.604 | 0.272 | -0.025 |
| PC 36:1 | 11 | 0 | 0 | 0.231 | 0 | 0 | 0 | 0.147 | 0 |
| PC 38:6 | 12 | 0.027 | 0.029 | 0.437 | -0.002 | 0.036 | 0.035 | 0.295 | 0.001 |
| PC 38:5 | 13 | 0.065 | 0.067 | 0.356 | -0.002 | 0.073 | 0.073 | 0.202 | 0 |
| PC 38:4 | 14 | 0.088 | 0.087 | 0.31 | 0.001 | 0.105 | 0.087 | 0.159 | 0.018 |
| PC 38:3 | 15 | 0.186 | 0.186 | 0.333 | 0 | 0.277 | 0.273 | 0.361 | 0.004 |
| PC 38:2 | 16 | 0.1 | 0.108 | 0.171 | -0.008 | 0.157 | 0.157 | 0.141 | 0 |
| PC 40:5 | 17 | 0.004 | 0.004 | 0.137 | 0 | 0.006 | 0.006 | 0.303 | 0 |
| PC 40:4 | 18 | 0.005 | 0.004 | 0.109 | 0.001 | 0.008 | 0.007 | 0.228 | 0.001 |
| PC 40:3 | 19 | 0.069 | 0.076 | 0.309 | -0.007 | 0.18 | 0.174 | 0.289 | 0.006 |
| PC 40:2 | 20 | 0.091 | 0.091 | 0.393 | 0 | 0.161 | 0.161 | 0.06 | 0 |
| **Total PC** |  | 38.196 | 37.83 | 0.12 | 0.366 | 39.564 | 39.762 | 0.409 | -0.198 |
| PE 32:3 | 21 | 0.032 | 0.045 | **0.02** | -0.013 | 0.059 | 0.055 | 0.324 | 0.004 |
| PE 32:2 | 22 | 0.043 | 0.059 | 0.189 | -0.016 | 0.075 | 0.076 | 0.415 | -0.001 |
| PE 32:1 | 23 | 0.02 | 0.019 | 0.477 | 0.001 | 0.044 | 0.013 | 0.162 | 0.031 |
| PE 32:0 | 24 | 0.006 | 0.007 | 0.467 | -0.001 | 0.002 | 0.003 | 0.321 | -0.001 |
| PE 34:4 | 25 | 0.043 | 0.053 | 0.125 | -0.01 | 0.063 | 0.05 | 0.085 | 0.013 |
| PE 34:3 | 26 | 10.014 | 9.408 | 0.224 | 0.606 | 10.485 | 9.664 | 0.232 | 0.821 |
| PE 34:2 | 27 | 8.726 | 8.629 | 0.221 | 0.097 | 9.285 | 8.032 | 0.273 | 1.253 |
| PE 34:1 | 28 | 0.159 | 0.137 | 0.293 | 0.022 | 0.09 | 0.063 | 0.328 | 0.027 |
| PE 36:6 | 29 | 2.836 | 2.649 | 0.157 | 0.187 | 2.446 | 2.345 | **0.038** | 0.101 |
| PE 36:5 | 30 | 5.347 | 5.379 | **0.046** | -0.032 | 5.044 | 4.589 | 0.127 | 0.455 |
| PE 36:4 | 31 | 2.61 | 3.098 | 0.122 | -0.488 | 2.664 | 2.547 | 0.166 | 0.117 |
| PE 36:3 | 32 | 1.026 | 1.054 | 0.203 | -0.028 | 1.019 | 0.98 | 0.491 | 0.039 |
| PE 36:2 | 33 | 0.72 | 0.768 | 0.175 | -0.048 | 0.782 | 0.728 | 0.374 | 0.054 |
| PE 36:1 | 34 | 0.01 | 0.008 | 0.243 | 0.002 | 0.014 | 0.01 | 0.186 | 0.004 |
| PE 38:6 | 35 | 0.015 | 0.016 | 0.32 | -0.001 | 0.016 | 0.016 | 0.345 | 0 |
| PE 38:5 | 36 | 0.024 | 0.024 | 0.41 | 0 | 0.029 | 0.026 | 0.37 | 0.003 |
| PE 38:4 | 37 | 0.028 | 0.028 | 0.32 | 0 | 0.033 | 0.028 | 0.226 | 0.005 |
| PE 38:3 | 38 | 0.106 | 0.104 | 0.347 | 0.002 | 0.144 | 0.142 | 0.397 | 0.002 |
| PE 38:2 | 39 | 0.118 | 0.109 | 0.084 | 0.009 | 0.144 | 0.145 | 0.178 | -0.001 |
| PE 40:3 | 40 | 0.12 | 0.12 | 0.358 | 0 | 0.215 | 0.211 | 0.393 | 0.004 |
| PE 40:2 | 41 | 0.212 | 0.21 | 0.466 | 0.002 | 0.352 | 0.325 | 0.437 | 0.027 |
| PE 42:3 | 42 | 0.266 | 0.245 | 0.202 | 0.021 | 0.253 | 0.229 | 0.061 | 0.024 |
| PE 42:2 | 43 | 0.356 | 0.349 | 0.38 | 0.007 | 0.317 | 0.288 | 0.304 | 0.029 |
| **Total PE** |  | 32.838 | 32.519 | 0.494 | 0.319 | 33.578 | 30.566 | **0.002** | 3.012 |
| PS 34:4 | 44 | 0 | 0 | 0.344 | 0 | 0 | 0 | 0.275 | 0 |
| PS 34:3 | 45 | 0.125 | 0.128 | 0.478 | -0.003 | 0.13 | 0.119 | 0.251 | 0.011 |
| PS 34:2 | 46 | 0.104 | 0.117 | **0.02** | -0.013 | 0.107 | 0.1 | 0.415 | 0.007 |
| PS 34:1 | 47 | 0.001 | 0.001 | 0.392 | 0 | 0 | 0.001 | 0.29 | -0.001 |
| PS 36:6 | 48 | 0.002 | 0.002 | 0.452 | 0 | 0.001 | 0.002 | 0.226 | -0.001 |
| PS 36:5 | 49 | 0.004 | 0.005 | 0.33 | -0.001 | 0.003 | 0.003 | 0.495 | 0 |
| PS 36:4 | 50 | 0.005 | 0.006 | 0.311 | -0.001 | 0.005 | 0.004 | 0.086 | 0.001 |
| PS 36:3 | 51 | 0.048 | 0.052 | **0.047** | -0.004 | 0.038 | 0.036 | 0.17 | 0.002 |
| PS 36:2 | 52 | 0.038 | 0.044 | 0.228 | -0.006 | 0.031 | 0.03 | 0.216 | 0.001 |
| PS 36:1 | 53 | 0 | 0 | 0.487 | 0 | 0 | 0 | 0.099 | 0 |
| PS 38:6 | 54 | 0 | 0 | 0.411 | 0 | 0 | 0 | 0.211 | 0 |
| PS 38:5 | 55 | 0 | 0.001 | 0.419 | -0.001 | 0 | 0.001 | 0.231 | -0.001 |
| PS 38:4 | 56 | 0.003 | 0.003 | 0.205 | 0 | 0.002 | 0.002 | 0.443 | 0 |
| PS 38:3 | 57 | 0.073 | 0.076 | 0.389 | -0.003 | 0.046 | 0.045 | 0.226 | 0.001 |
| PS 38:2 | 58 | 0.061 | 0.075 | 0.167 | -0.014 | 0.047 | 0.047 | 0.489 | 0 |
| PS 38:1 | 59 | 0.001 | 0 | 0.248 | 0.001 | 0 | 0 | 0.211 | 0 |
| PS 40:4 | 60 | 0.002 | 0.003 | 0.179 | -0.001 | 0.001 | 0.001 | 0.265 | 0 |
| PS 40:3 | 61 | 0.16 | 0.172 | 0.327 | -0.012 | 0.155 | 0.157 | 0.351 | -0.002 |
| PS 40:2 | 62 | 0.139 | 0.165 | 0.166 | -0.026 | 0.151 | 0.139 | 0.376 | 0.012 |
| PS 40:1 | 63 | 0.001 | 0 | 0.248 | 0.001 | 0 | 0 | 0.382 | 0 |
| PS 42:4 | 64 | 0.001 | 0.001 | 0.329 | 0 | 0.001 | 0.002 | 0.486 | -0.001 |
| PS 42:3 | 65 | 0.335 | 0.317 | 0.482 | 0.018 | 0.375 | 0.331 | 0.494 | 0.044 |
| PS 42:2 | 66 | 0.201 | 0.209 | 0.179 | -0.008 | 0.235 | 0.21 | 0.393 | 0.025 |
| PS 42:1 | 67 | 0.004 | 0.001 | **0.036** | 0.003 | 0.004 | 0.003 | 0.376 | 0.001 |
| PS 44:3 | 68 | 0.022 | 0.02 | 0.389 | 0.002 | 0.019 | 0.018 | 0.38 | 0.001 |
| PS 44:2 | 69 | 0.02 | 0.017 | 0.493 | 0.003 | 0.02 | 0.014 | 0.38 | 0.006 |
| **Total PS** |  | 1.35 | 1.414 | 0.3 | -0.064 | 1.373 | 1.267 | **0.025** | 0.106 |
| DGDG 34:6 | 70 | 0.002 | 0.002 | 0.463 | 0 | 0.001 | 0.007 | 0.232 | -0.006 |
| DGDG 34:5 | 71 | 0.001 | 0 | 0.248 | 0.001 | 0.001 | 0.001 | 0.211 | 0 |
| DGDG 34:4 | 72 | 0.019 | 0.021 | 0.185 | -0.002 | 0.01 | 0.017 | 0.242 | -0.007 |
| DGDG 34:3 | 73 | 0.681 | 0.658 | 0.139 | 0.023 | 0.245 | 0.287 | 0.368 | -0.042 |
| DGDG 34:2 | 74 | 0.121 | 0.114 | 0.189 | 0.007 | 0.01 | 0.015 | 0.353 | -0.005 |
| DGDG 34:1 | 75 | 0.012 | 0.007 | 0.082 | 0.005 | 0.001 | 0.007 | 0.211 | -0.006 |
| DGDG 36:6 | 76 | 2.568 | 2.62 | 0.432 | -0.052 | 1.363 | 1.496 | 0.347 | -0.133 |
| DGDG 36:5 | 77 | 0.188 | 0.207 | 0.172 | -0.019 | 0.052 | 0.053 | 0.142 | -0.001 |
| DGDG 36:4 | 78 | 0.177 | 0.191 | 0.31 | -0.014 | 0.076 | 0.082 | 0.293 | -0.006 |
| DGDG 36:3 | 79 | 0.259 | 0.264 | 0.486 | -0.005 | 0.141 | 0.158 | 0.432 | -0.017 |
| DGDG 36:2 | 80 | 0.023 | 0.028 | 0.5 | -0.005 | 0.002 | 0.006 | 0.497 | -0.004 |
| DGDG 38:6 | 81 | 0.001 | 0.002 | 0.092 | -0.001 | 0.001 | 0.004 | 0.235 | -0.003 |
| DGDG 38:5 | 82 | 0 | 0.001 | 0.48 | -0.001 | 0 | 0 | 0.356 | 0 |
| DGDG 38:4 | 83 | 0.002 | 0.002 | 0.393 | 0 | 0 | 0.001 | 0.266 | -0.001 |
| DGDG 38:3 | 84 | 0.003 | 0.004 | 0.396 | -0.001 | 0.001 | 0.003 | 0.282 | -0.002 |
| **Total DGDG** |  | 4.059 | 4.121 | 0.383 | -0.062 | 1.904 | 2.137 | **0.043** | -0.233 |
| PA 32:0 | 85 | 0.005 | 0.006 | 0.406 | -0.001 | 0.012 | 0.013 | 0.425 | -0.001 |
| PA 34:6 | 86 | 0 | 0.001 | **0.06** | -0.001 | 0 | 0.004 | 0.221 | -0.004 |
| PA 34:5 | 87 | 0 | 0 | 0.248 | 0 | 0 | 0 | 0.167 | 0 |
| PA 34:4 | 88 | 0.002 | 0.003 | **0.049** | -0.001 | 0.002 | 0.002 | 0.452 | 0 |
| PA 34:3 | 89 | 0.353 | 0.392 | 0.287 | -0.039 | 0.35 | 0.374 | 0.101 | -0.024 |
| PA 34:2 | 90 | 0.261 | 0.299 | 0.289 | -0.038 | 0.246 | 0.265 | 0.343 | -0.019 |
| PA 34:1 | 91 | 0.008 | 0.003 | 0.174 | 0.005 | 0.005 | 0.007 | 0.385 | -0.002 |
| PA 36:6 | 92 | 0.148 | 0.169 | 0.334 | -0.021 | 0.104 | 0.103 | 0.215 | 0.001 |
| PA 36:5 | 93 | 0.239 | 0.277 | 0.345 | -0.038 | 0.17 | 0.158 | 0.332 | 0.012 |
| PA 36:4 | 94 | 0.137 | 0.165 | 0.3 | -0.028 | 0.095 | 0.103 | 0.298 | -0.008 |
| PA 36:3 | 95 | 0.05 | 0.064 | 0.232 | -0.014 | 0.049 | 0.047 | 0.454 | 0.002 |
| PA 36:2 | 96 | 0.036 | 0.036 | 0.439 | 0 | 0.03 | 0.034 | 0.195 | -0.004 |
| **Total PA** |  | 1.24 | 1.415 | 0.415 | -0.175 | 1.063 | 1.11 | **0.045** | -0.047 |
| MGDG 34:6 | 97 | 0.001 | 0.012 | 0.248 | -0.011 | 0 | 0.096 | 0.211 | -0.096 |
| MGDG 34:5 | 98 | 0.001 | 0.005 | 0.169 | -0.004 | 0.003 | 0.009 | 0.189 | -0.006 |
| MGDG 34:4 | 99 | 0.021 | 0.024 | 0.217 | -0.003 | 0.018 | 0.024 | 0.189 | -0.006 |
| MGDG 34:3 | 100 | 0.12 | 0.126 | 0.415 | -0.006 | 0.069 | 0.085 | 0.426 | -0.016 |
| MGDG 34:2 | 101 | 0.014 | 0.012 | 0.323 | 0.002 | 0.002 | 0.004 | 0.293 | -0.002 |
| MGDG 34:1 | 102 | 0.006 | 0.001 | **0.028** | 0.005 | 0.001 | 0 | 0.254 | 0.001 |
| MGDG 36:6 | 103 | 3.792 | 3.871 | 0.39 | -0.079 | 2.714 | 2.931 | 0.432 | -0.217 |
| MGDG 36:5 | 104 | 0.234 | 0.24 | 0.254 | -0.006 | 0.119 | 0.112 | 0.129 | 0.007 |
| MGDG 36:4 | 105 | 0.085 | 0.094 | 0.321 | -0.009 | 0.051 | 0.054 | 0.168 | -0.003 |
| MGDG 36:3 | 106 | 0.025 | 0.028 | 0.088 | -0.003 | 0.015 | 0.021 | 0.198 | -0.006 |
| MGDG 36:2 | 107 | 0.003 | 0.003 | 0.304 | 0 | 0.002 | 0.001 | 0.398 | 0.001 |
| MGDG 36:1 | 108 | 0.001 | 0 | **0.037** | 0.001 | 0 | 0 | 0.136 | 0 |
| MGDG 38:6 | 109 | 0.002 | 0.002 | 0.116 | 0 | 0.001 | 0.002 | 0.258 | -0.001 |
| MGDG 38:5 | 110 | 0.001 | 0 | **0.01** | 0.001 | 0 | 0.001 | 0.227 | -0.001 |
| MGDG 38:4 | 111 | 0 | 0 | 0.393 | 0 | 0 | 0.001 | 0.133 | -0.001 |
| MGDG 38:3 | 112 | 0 | 0 | 0.081 | 0 | 0 | 0 | 0.212 | 0 |
| **Total MGDG** |  | 4.306 | 4.419 | 0.431 | -0.113 | 2.994 | 3.341 | **0.041** | -0.347 |
| PG 32:1 | 113 | 0.004 | 0.005 | 0.455 | -0.001 | 0.007 | 0.004 | 0.255 | 0.003 |
| PG 32:0 | 114 | 0.789 | 0.7 | 0.272 | 0.089 | 0.468 | 0.462 | 0.28 | 0.006 |
| PG 34:3 | 115 | 0.783 | 0.687 | 0.306 | 0.096 | 0.658 | 0.641 | 0.406 | 0.017 |
| PG 34:2 | 116 | 0.431 | 0.386 | 0.209 | 0.045 | 0.375 | 0.355 | 0.439 | 0.02 |
| PG 34:1 | 117 | 0.078 | 0.08 | 0.335 | -0.002 | 0.061 | 0.049 | 0.27 | 0.012 |
| PG 34:0 | 118 | 0.071 | 0.073 | 0.278 | -0.002 | 0.053 | 0.054 | 0.183 | -0.001 |
| PG 36:6 | 119 | 0.014 | 0.012 | 0.445 | 0.002 | 0.014 | 0.014 | 0.362 | 0 |
| PG 36:5 | 120 | 0.018 | 0.017 | 0.381 | 0.001 | 0.017 | 0.019 | **0.038** | -0.002 |
| PG 36:4 | 121 | 0.011 | 0.009 | 0.281 | 0.002 | 0.009 | 0.007 | 0.366 | 0.002 |
| PG 36:3 | 122 | 0.025 | 0.022 | **0.041** | 0.003 | 0.02 | 0.02 | 0.27 | 0 |
| PG 36:2 | 123 | 0.021 | 0.021 | 0.377 | 0 | 0.018 | 0.018 | 0.159 | 0 |
| PG 36:1 | 124 | 0 | 0 | 0.161 | 0 | 0.001 | 0 | 0.056 | 0.001 |
| **Total PG** |  | 2.249 | 2.016 | 0.244 | 0.233 | 1.704 | 1.648 | 0.296 | 0.056 |
| PI 32:3 | 125 | 0.01 | 0.015 | 0.111 | -0.005 | 0.027 | 0.028 | 0.105 | -0.001 |
| PI 32:2 | 126 | 0.013 | 0.016 | 0.227 | -0.003 | 0.024 | 0.026 | 0.245 | -0.002 |
| PI 32:1 | 127 | 0.018 | 0.021 | 0.159 | -0.003 | 0.022 | 0.03 | 0.133 | -0.008 |
| PI 32:0 | 128 | 0.101 | 0.114 | **0.042** | -0.013 | 0.124 | 0.147 | 0.102 | -0.023 |
| PI 34:4 | 129 | 0.019 | 0.03 | **0.022** | -0.011 | 0.024 | 0.037 | 0.234 | -0.013 |
| PI 34:3 | 130 | 9.039 | 9.216 | 0.462 | -0.177 | 10.117 | 11.675 | 0.368 | -1.558 |
| PI 34:2 | 131 | 4.102 | 4.311 | 0.21 | -0.209 | 4.677 | 5.203 | 0.447 | -0.526 |
| PI 34:1 | 132 | 0.104 | 0.103 | 0.235 | 0.001 | 0.099 | 0.09 | 0.339 | 0.009 |
| PI 36:6 | 133 | 0.268 | 0.253 | **0.045** | 0.015 | 0.344 | 0.371 | 0.386 | -0.027 |
| PI 36:5 | 134 | 0.384 | 0.394 | 0.407 | -0.01 | 0.489 | 0.533 | 0.179 | -0.044 |
| PI 36:4 | 135 | 0.308 | 0.337 | 0.129 | -0.029 | 0.331 | 0.363 | **0.046** | -0.032 |
| PI 36:3 | 136 | 0.712 | 0.737 | 0.338 | -0.025 | 0.778 | 0.859 | 0.446 | -0.081 |
| PI 36:2 | 137 | 0.429 | 0.466 | 0.356 | -0.037 | 0.502 | 0.551 | 0.238 | -0.049 |
| PI 36:1 | 138 | 0.01 | 0.009 | 0.405 | 0.001 | 0.017 | 0.014 | 0.36 | 0.003 |
| **Total PI** |  | 15.517 | 16.022 | 0.176 | -0.505 | 17.573 | 19.929 | 0.102 | -2.356 |
| LPG 16:1 | 139 | 0.008 | 0.009 | 0.453 | -0.001 | 0.002 | 0 | **0.042** | 0.002 |
| LPG 16:0 | 140 | 0 | 0.005 | **0.006** | -0.005 | 0.002 | 0.001 | 0.211 | 0.001 |
| LPG 18:3 | 141 | 0 | 0.001 | 0.125 | -0.001 | 0 | 0 | 0.167 | 0 |
| LPG 18:2 | 142 | 0 | 0 | 0.168 | 0 | 0 | 0.004 | 0.213 | -0.004 |
| LPG 18:1 | 143 | 0 | 0 | 0.375 | 0 | 0 | 0.001 | 0.211 | -0.001 |
| **Total LPG** |  | 0.008 | 0.015 | 0.362 | -0.007 | 0.004 | 0.007 | 0.387 | -0.003 |
| LPC(16:1) | 144 | 0 | 0 | 0.485 | 0 | 0 | 0 | 0.481 | 0 |
| LPC(16:0) | 145 | 0.019 | 0.02 | 0.416 | -0.001 | 0.021 | 0.029 | **0.036** | -0.008 |
| LPC(18:3) | 146 | 0.026 | 0.023 | **0.034** | 0.003 | 0.023 | 0.024 | 0.255 | -0.001 |
| LPC(18:2) | 147 | 0.017 | 0.016 | 0.13 | 0.001 | 0.015 | 0.018 | **0.045** | -0.003 |
| LPC(18:1) | 148 | 0.003 | 0.001 | 0.098 | 0.002 | 0.001 | 0.001 | 0.055 | 0 |
| LPC(18:0) | 149 | 0.004 | 0.003 | 0.073 | 0.001 | 0.005 | 0.009 | 0.076 | -0.004 |
| **TOTAL LPC** |  | 0.07 | 0.062 | **0.027** | 0.008 | 0.066 | 0.08 | 0.072 | -0.014 |
| LPE(16:1) | 150 | 0 | 0 | 0.146 | 0 | 0 | 0 | 0.236 | 0 |
| LPE(16:0) | 151 | 0.089 | 0.087 | 0.495 | 0.002 | 0.098 | 0.085 | 0.234 | 0.013 |
| LPE(18:3) | 152 | 0.041 | 0.036 | 0.209 | 0.005 | 0.041 | 0.032 | 0.31 | 0.009 |
| LPE(18:2) | 153 | 0.037 | 0.042 | **0.043** | -0.005 | 0.039 | 0.035 | 0.389 | 0.004 |
| LPE(18:1) | 154 | 0.002 | 0.001 | 0.458 | 0.001 | 0.001 | 0.001 | 0.466 | 0 |
| **TOTAL LPE** |  | 0.168 | 0.167 | 0.324 | 0.001 | 0.179 | 0.153 | 0.289 | 0.026 |

**Supplementary Table 3:** **Amounts of polar lipid molecular species as affected by phosphate starvation.** Stripped roots and root hairs of 7-day-old seedling grown on modified Murashige and Skoog agar medium with phosphate sufficient (1 mM) and deficient (0 mM) conditions were used to analyze the lipids. Five biological replications were maintained for each experiment and the experiment was repeated twice. The lipids were analyzed by ESI-MS/MS and the intensities in each spectrum were normalized to those two internal standards of the same class; a signal of 1 is the same amount of intensity as 1 nmol of the standards. The data represents the average of 10 replicates. The data of phosphate sufficient and deficient conditions were compared via *t* test and the P values are reported. The P value <0.05 is presented in bold.

| **Lipid species (class and total acyl carbons: total carbon-carbon double bonds)** | **Compound Number** | Polar lipid molecular species composition (mol %)  (polar lipid molecular species as % (mol/mol) total polar lipid molecular species) | | | | | | | |
| --- | --- | --- | --- | --- | --- | --- | --- | --- | --- |
|  |  | **Stripped Root** | | | | **Root hair** | | | |
|  |  | **Pi**  **1 mM** | **Pi**  **0 mM** | **P value** | **Difference between**  **Pi 1 mM and**  **Pi 0 mM** | **Pi**  **1 mM** | **Pi**  **0 mM** | **Pi value** | **Difference between**  **Pi 1 mM and**  **Pi 0 mM** |
| PC 32:0 | 1 | 0.108 | 0.07 | 0.304 | 0.038 | 0.076 | 0.069 | 0.116 | 0.007 |
| PC 34:4 | 2 | 0.136 | 0.084 | 0.227 | 0.052 | 0.095 | 0.077 | 0.084 | 0.018 |
| PC 34:3 | 3 | 10.060 | 7.878 | 0.235 | 2.182 | 6.662 | 7.273 | 0.458 | -0.611 |
| PC 34:2 | 4 | 7.035 | 6.808 | 0.476 | 0.227 | 5.897 | 6.379 | 0.456 | -0.482 |
| PC 34:1 | 5 | 0.222 | 0.134 | 0.263 | 0.088 | 0.481 | 0.622 | 0.181 | -0.141 |
| PC 36:6 | 6 | 5.964 | 4.717 | 0.146 | 1.247 | 4.028 | 4.912 | 0.24 | -0.884 |
| PC 36:5 | 7 | 8.665 | 8.605 | 0.497 | 0.06 | 10.104 | 9.154 | 0.232 | 0.95 |
| PC 36:4 | 8 | 4.087 | 4.811 | 0.126 | -0.724 | 3.342 | 3.898 | 0.204 | -0.556 |
| PC 36:3 | 9 | 2.708 | 2.736 | 0.221 | -0.028 | 2.973 | 2.439 | **0.04** | 0.534 |
| PC 36:2 | 10 | 1.589 | 1.839 | 0.061 | -0.25 | 1.650 | 1.856 | 0.353 | -0.206 |
| PC 36:1 | 11 | 0 | 0 | 0.126 | 0 | 0.038 | 0.022 | 0.383 | 0.016 |
| PC 38:6 | 12 | 0.043 | 0.03 | 0.167 | 0.013 | 0.036 | 0.032 | 0.416 | 0.004 |
| PC 38:5 | 13 | 0.087 | 0.074 | 0.428 | 0.013 | 0.092 | 0.08 | 0.225 | 0.012 |
| PC 38:4 | 14 | 0.125 | 0.085 | 0.235 | 0.04 | 0.146 | 0.12 | **0.044** | 0.026 |
| PC 38:3 | 15 | 0.186 | 0.181 | 0.425 | 0.005 | 0.170 | 0.212 | 0.066 | -0.042 |
| PC 38:2 | 16 | 0.109 | 0.112 | 0.113 | -0.003 | 0.144 | 0.11 | 0.088 | 0.034 |
| PC 40:5 | 17 | 0.006 | 0.003 | 0.083 | 0.003 | 0.010 | 0.009 | **0.048** | 0.001 |
| PC 40:4 | 18 | 0.009 | 0.002 | 0.496 | 0.007 | 0.010 | 0.006 | 0.087 | 0.004 |
| PC 40:3 | 19 | 0.049 | 0.028 | 0.218 | 0.021 | 0.081 | 0.069 | 0.081 | 0.012 |
| PC 40:2 | 20 | 0.081 | 0.056 | 0.245 | 0.025 | 0.090 | 0.115 | 0.145 | -0.025 |
| **Total PC** |  | 41.266 | 38.251 | 0.301 | 3.015 | 36.125 | 37.454 | 0.365 | -1.329 |
| PE 32:3 | 21 | 0.022 | 0.019 | 0.082 | 0.003 | 0.026 | 0.022 | 0.383 | 0.004 |
| PE 32:2 | 22 | 0.043 | 0.024 | 0.101 | 0.019 | 0.047 | 0.033 | 0.106 | 0.014 |
| PE 32:1 | 23 | 0.011 | 0.002 | **0.023** | 0.009 | 0.019 | 0.009 | **0.044** | 0.01 |
| PE 32:0 | 24 | 0.005 | 0.004 | 0.177 | 0.001 | 0.005 | 0.002 | 0.253 | 0.003 |
| PE 34:4 | 25 | 0.035 | 0.033 | 0.12 | 0.002 | 0.053 | 0.042 | **0.044** | 0.011 |
| PE 34:3 | 26 | 8.018 | 6.755 | **0.044** | 1.263 | 9.781 | 8.744 | 0.192 | 1.037 |
| PE 34:2 | 27 | 6.198 | 5.772 | 0.22 | 0.426 | 8.068 | 8.421 | 0.202 | -0.353 |
| PE 34:1 | 28 | 0 | 0.049 | 0.449 | -0.049 | 0.295 | 0.131 | 0.207 | 0.164 |
| PE 36:6 | 29 | 2.727 | 1.797 | **0.002** | 0.93 | 3.145 | 2.92 | 0.278 | 0.225 |
| PE 36:5 | 30 | 4.790 | 4.445 | 0.386 | 0.345 | 7.279 | 6.265 | 0.303 | 1.014 |
| PE 36:4 | 31 | 3.219 | 3.696 | 0.123 | -0.477 | 2.554 | 2.517 | 0.21 | 0.037 |
| PE 36:3 | 32 | 1.130 | 1.014 | 0.387 | 0.116 | 1.036 | 0.994 | 0.377 | 0.042 |
| PE 36:2 | 33 | 0.694 | 0.832 | 0.216 | -0.138 | 0.787 | 0.876 | 0.342 | -0.089 |
| PE 36:1 | 34 | 0.033 | 0 | 0.211 | 0.033 | 0.016 | 0.014 | 0.358 | 0.002 |
| PE 38:6 | 35 | 0.016 | 0.015 | 0.407 | 0.001 | 0.016 | 0.019 | 0.282 | -0.003 |
| PE 38:5 | 36 | 0.031 | 0.032 | 0.323 | -0.001 | 0.030 | 0.031 | 0.477 | -0.001 |
| PE 38:4 | 37 | 0.026 | 0.034 | 0.147 | -0.008 | 0.026 | 0.038 | 0.064 | -0.012 |
| PE 38:3 | 38 | 0.077 | 0.096 | 0.153 | -0.019 | 0.124 | 0.135 | 0.241 | -0.011 |
| PE 38:2 | 39 | 0.106 | 0.072 | 0.483 | 0.034 | 0.144 | 0.156 | 0.476 | -0.012 |
| PE 40:3 | 40 | 0.067 | 0.043 | **0.043** | 0.024 | 0.130 | 0.156 | 0.149 | -0.026 |
| PE 40:2 | 41 | 0.130 | 0.095 | **0.045** | 0.035 | 0.266 | 0.27 | 0.374 | -0.004 |
| PE 42:3 | 42 | 0.190 | 0.103 | **0.045** | 0.087 | 0.285 | 0.259 | 0.15 | 0.026 |
| PE 42:2 | 43 | 0.352 | 0.252 | 0.3 | 0.1 | 0.332 | 0.349 | 0.074 | -0.017 |
| **Total PE** |  | 27.918 | 25.184 | **0.045** | 2.734 | 34.466 | 32.402 | **0.041** | 2.064 |
| PS 34:4 | 44 | 0 | 0.001 | 0.211 | -0.001 | 0.005 | 0 | 0.211 | 0.005 |
| PS 34:3 | 45 | 0.110 | 0.076 | **0.031** | 0.034 | 0.147 | 0.157 | 0.063 | -0.01 |
| PS 34:2 | 46 | 0.126 | 0.086 | **0.042** | 0.04 | 0.090 | 0.159 | 0.07 | -0.069 |
| PS 34:1 | 47 | 0 | 0 | 0.211 | 0 | 0.003 | 0.002 | 0.449 | 0.001 |
| PS 36:6 | 48 | 0 | 0 | 0.311 | 0 | 0.002 | 0.003 | 0.333 | -0.001 |
| PS 36:5 | 49 | 0.001 | 0.007 | 0.26 | -0.006 | 0.018 | 0.006 | 0.234 | 0.012 |
| PS 36:4 | 50 | 0.011 | 0.009 | 0.115 | 0.002 | 0.007 | 0.005 | 0.376 | 0.002 |
| PS 36:3 | 51 | 0.078 | 0.064 | 0.143 | 0.014 | 0.048 | 0.056 | 0.339 | -0.008 |
| PS 36:2 | 52 | 0.071 | 0.069 | 0.198 | 0.002 | 0.022 | 0.042 | 0.213 | -0.02 |
| PS 36:1 | 53 | 0 | 0 | 0.153 | 0 | 0 | 0.002 | 0.063 | -0.002 |
| PS 38:6 | 54 | 0.001 | 0 | 0.221 | 0.001 | 0 | 0 | 0.211 | 0 |
| PS 38:5 | 55 | 0 | 0.001 | 0.3 | -0.001 | 0 | 0 | 0.131 | 0 |
| PS 38:4 | 56 | 0.003 | 0.003 | 0.131 | 0 | 0.0018 | 0.004 | 0.093 | -0.0022 |
| PS 38:3 | 57 | 0.105 | 0.086 | 0.082 | 0.019 | 0.066 | 0.102 | 0.199 | -0.036 |
| PS 38:2 | 58 | 0.118 | 0.118 | 0.311 | 0 | 0.077 | 0.1 | 0.225 | -0.023 |
| PS 38:1 | 59 | 0 | 0.001 | 0.211 | -0.001 | 0 | 0 | 0.161 | 0 |
| PS 40:4 | 60 | 0 | 0.001 | 0.211 | -0.001 | 0.001 | 0.001 | 0.2 | 0 |
| PS 40:3 | 61 | 0.233 | 0.113 | 0.082 | 0.12 | 0.182 | 0.19 | 0.372 | -0.008 |
| PS 40:2 | 62 | 0.252 | 0.159 | 0.456 | 0.093 | 0.086 | 0.167 | 0.236 | -0.081 |
| PS 40:1 | 63 | 0 | 0 | 0.141 | 0 | 0 | 0 | 0.198 | 0 |
| PS 42:4 | 64 | 0 | 0 | 0.131 | 0 | 0.0003 | 0 | 0.425 | 0.0003 |
| PS 42:3 | 65 | 0.409 | 0.242 | 0.12 | 0.167 | 0.377 | 0.322 | 0.232 | 0.055 |
| PS 42:2 | 66 | 0.159 | 0.202 | 0.292 | -0.043 | 0.164 | 0.203 | 0.372 | -0.039 |
| PS 42:1 | 67 | 0 | 0 | 0.258 | 0 | 0 | 0.001 | 0.2 99 | -0.001 |
| PS 44:3 | 68 | 0.008 | 0.011 | 0.23 | -0.003 | 0.001 | 0.013 | 0.081 | -0.012 |
| PS 44:2 | 69 | 0.014 | 0.017 | 0.231 | -0.003 | 0.112 | 0.011 | 0.209 | 0.101 |
| **Total PS** |  | 1.697 | 1.266 | **0.012** | 0.431 | 1.412 | 1.548 | 0.465 | -0.136 |
| DGDG 34:6 | 70 | 0.002 | 0.006 | 0.298 | -0.004 | 0 | 0 | 0.179 | 0 |
| DGDG 34:5 | 71 | 0 | 0 | 0.321 | 0 | 0 | 0.001 | 0.211 | -0.001 |
| DGDG 34:4 | 72 | 0.040 | 0.027 | 0.485 | 0.013 | 0.0126 | 0.011 | 0.077 | 0.0016 |
| DGDG 34:3 | 73 | 0.895 | 0.742 | 0.22 | 0.153 | 0.368 | 0.509 | 0.1 | -0.141 |
| DGDG 34:2 | 74 | 0.098 | 0.08 | 0.434 | 0.018 | 0.046 | 0.072 | 0.377 | -0.026 |
| DGDG 34:1 | 75 | 0.027 | 0.016 | 0.384 | 0.011 | 0.013 | 0.01 | 0.256 | 0.003 |
| DGDG 36:6 | 76 | 2.497 | 3.677 | **0.045** | -1.18 | 1.882 | 2.029 | **0.032** | -0.147 |
| DGDG 36:5 | 77 | 0.239 | 0.391 | **0.044** | -0.152 | 0.190 | 0.131 | 0.268 | 0.059 |
| DGDG 36:4 | 78 | 0.144 | 0.231 | **0.053** | -0.087 | 0.090 | 0.08 | 0.054 | 0.01 |
| DGDG 36:3 | 79 | 0.253 | 0.423 | 0.158 | -0.17 | 0.128 | 0.182 | 0.069 | -0.054 |
| DGDG 36:2 | 80 | 0.007 | 0.056 | 0.071 | -0.049 | 0.009 | 0.018 | 0.469 | -0.009 |
| DGDG 38:6 | 81 | 0.001 | 0.014 | 0.321 | -0.013 | 0.005 | 0.003 | 0.155 | 0.002 |
| DGDG 38:5 | 82 | 0 | 0 | 0.131 | 0 | 0 | 0 | 0.258 | 0 |
| DGDG 38:4 | 83 | 0 | 0.001 | 0.176 | -0.001 | 0 | 0 | 0.356 | 0 |
| DGDG 38:3 | 84 | 0 | 0.003 | 0.079 | -0.003 | 0 | 0.001 | 0.333 | -0.001 |
| **Total DGDG** |  | 4.204 | 5.667 | **0.041** | -1.463 | 2.743 | 3.047 | **0.006** | -0.304 |
| PA 32:0 | 85 | 0 | 0.003 | 0.226 | -0.003 | 0.006 | 0.009 | 0.183 | -0.003 |
| PA 34:6 | 86 | 0 | 0 | 0.151 | 0 | 0 | 0 | 0.211 | 0 |
| PA 34:5 | 87 | 0 | 0 | 0.131 | 0 | 0.010 | 0 | 0.205 | 0.01 |
| PA 34:4 | 88 | 0.011 | 0.004 | 0.418 | 0.007 | 0.0007 | 0.004 | **0.04** | -0.0033 |
| PA 34:3 | 89 | 0.698 | 0.452 | 0.053 | 0.246 | 0.482 | 0.697 | 0.124 | -0.215 |
| PA 34:2 | 90 | 0.360 | 0.456 | 0.379 | -0.096 | 0.339 | 0.528 | **0.044** | -0.189 |
| PA 34:1 | 91 | 0 | 0.005 | 0.334 | -0.005 | 0.002 | 0.004 | 0.291 | -0.002 |
| PA 36:6 | 92 | 0.263 | 0.184 | 0.093 | 0.079 | 0.160 | 0.278 | **0.045** | -0.118 |
| PA 36:5 | 93 | 0.412 | 0.55 | 0.322 | -0.138 | 0.301 | 0.524 | **0.025** | -0.223 |
| PA 36:4 | 94 | 0.222 | 0.36 | 0.427 | -0.138 | 0.133 | 0.263 | 0.109 | -0.13 |
| PA 36:3 | 95 | 0.067 | 0.071 | 0.183 | -0.004 | 0.061 | 0.104 | 0.117 | -0.043 |
| PA 36:2 | 96 | 0.045 | 0.058 | 0.252 | -0.013 | 0.050 | 0.064 | 0.13 | -0.014 |
| **Total PA** |  | 2.078 | 2.143 | 0.195 | -0.065 | 1.544 | 2.476 | **0.044** | -0.932 |
| MGDG 34:6 | 97 | 0.002 | 0.017 | 0.1 | -0.015 | 0.008 | 0.002 | 0.181 | 0.006 |
| MGDG 34:5 | 98 | 0.005 | 0.01 | 0.095 | -0.005 | 0.007 | 0.004 | 0.193 | 0.003 |
| MGDG 34:4 | 99 | 0.026 | 0.03 | 0.497 | -0.004 | 0.022 | 0.019 | 0.439 | 0.003 |
| MGDG 34:3 | 100 | 0.144 | 0.143 | 0.151 | 0.001 | 0.144 | 0.136 | 0.235 | 0.008 |
| MGDG 34:2 | 101 | 0.007 | 0.025 | 0.173 | -0.018 | 0.006 | 0.015 | **0.048** | -0.009 |
| MGDG 34:1 | 102 | 0.005 | 0.005 | 0.173 | 0 | 0.002 | 0.004 | 0.149 | -0.002 |
| MGDG 36:6 | 103 | 4.074 | 5.699 | 0.118 | -1.625 | 3.136 | 3.143 | 0.433 | -0.007 |
| MGDG 36:5 | 104 | 0.170 | 0.426 | **0.004** | -0.256 | 0.240 | 0.207 | 0.232 | 0.033 |
| MGDG 36:4 | 105 | 0.100 | 0.1 | 0.157 | 0 | 0.070 | 0.064 | 0.259 | 0.006 |
| MGDG 36:3 | 106 | 0.033 | 0.049 | **0.043** | -0.016 | 0.025 | 0.025 | 0.369 | 0 |
| MGDG 36:2 | 107 | 0 | 0.002 | 0.474 | -0.002 | 0.003 | 0.006 | **0.048** | -0.003 |
| MGDG 36:1 | 108 | 0.007 | 0 | 0.055 | 0.007 | 0.001 | 0.001 | 0.385 | 0 |
| MGDG 38:6 | 109 | 0.013 | 0.015 | 0.298 | -0.002 | 0.006 | 0.02 | 0.15 | -0.014 |
| MGDG 38:5 | 110 | 0.002 | 0 | 0.127 | 0.002 | 0.001 | 0.001 | 0.211 | 0 |
| MGDG 38:4 | 111 | 0.004 | 0 | 0.238 | 0.004 | 0 | 0.001 | 0.211 | -0.001 |
| MGDG 38:3 | 112 | 0.0004 | 0 | 0.405 | 0.0004 | 0.002 | 0.001 | 0.178 | 0.001 |
| **Total MGDG** |  | 4.592 | 6.521 | **0.047** | -1.929 | 3.672 | 3.65 | 0.463 | 0.022 |
| PG 32:1 | 113 | 0.0132 | 0 | 0.464 | 0.0132 | 0.007 | 0.006 | **0.015** | 0.001 |
| PG 32:0 | 114 | 0.703 | 0.599 | **0.03** | 0.104 | 0.938 | 0.913 | 0.49 | 0.025 |
| PG 34:3 | 115 | 0.807 | 0.544 | **0.017** | 0.263 | 0.825 | 0.983 | 0.143 | -0.158 |
| PG 34:2 | 116 | 0.432 | 0.402 | 0.278 | 0.03 | 0.683 | 0.598 | 0.361 | 0.085 |
| PG 34:1 | 117 | 0.055 | 0.047 | 0.072 | 0.008 | 0.090 | 0.144 | 0.127 | -0.054 |
| PG 34:0 | 118 | 0.074 | 0.092 | 0.051 | -0.018 | 0.080 | 0.083 | 0.279 | -0.003 |
| PG 36:6 | 119 | 0.019 | 0.007 | **0.045** | 0.012 | 0.015 | 0.013 | 0.454 | 0.002 |
| PG 36:5 | 120 | 0.032 | 0.013 | 0.247 | 0.019 | 0.023 | 0.029 | 0.091 | -0.006 |
| PG 36:4 | 121 | 0.014 | 0.011 | 0.411 | 0.003 | 0.022 | 0.02 | 0.21 | 0.002 |
| PG 36:3 | 122 | 0.024 | 0.003 | 0.472 | 0.021 | 0.033 | 0.032 | 0.136 | 0.001 |
| PG 36:2 | 123 | 0.031 | 0.023 | 0.435 | 0.008 | 0.032 | 0.102 | 0.061 | -0.07 |
| PG 36:1 | 124 | 0.007 | 0 | 0.055 | 0.007 | 0.005 | 0.007 | 0.21 | -0.002 |
| **Total PG** |  | 2.214 | 1.742 | 0.135 | 0.472 | 2.756 | 2.93 | 0.167 | -0.174 |
| PI 32:3 | 125 | 0.006 | 0.004 | **0.048** | 0.002 | 0.005 | 0.006 | 0.156 | -0.001 |
| PI 32:2 | 126 | 0.004 | 0.012 | 0.331 | -0.008 | 0.021 | 0.007 | **0.041** | 0.014 |
| PI 32:1 | 127 | 0.036 | 0.01 | 0.215 | 0.026 | 0.008 | 0.012 | 0.303 | -0.004 |
| PI 32:0 | 128 | 0.068 | 0.093 | 0.131 | -0.025 | 0.070 | 0.075 | 0.228 | -0.005 |
| PI 34:4 | 129 | 0.014 | 0.021 | 0.372 | -0.007 | 0.021 | 0.015 | 0.167 | 0.006 |
| PI 34:3 | 130 | 8.768 | 10.35 | 0.346 | -1.582 | 8.287 | 8.437 | 0.261 | -0.15 |
| PI 34:2 | 131 | 3.428 | 5.598 | **0.048** | -2.17 | 4.933 | 4.541 | 0.204 | 0.392 |
| PI 34:1 | 132 | 0.181 | 0 | 0.229 | 0.181 | 0.051 | 0.123 | 0.164 | -0.072 |
| PI 36:6 | 133 | 0.261 | 0.221 | **0.036** | 0.04 | 0.315 | 0.325 | 0.461 | -0.01 |
| PI 36:5 | 134 | 0.395 | 0.354 | 0.276 | 0.041 | 0.546 | 0.586 | 0.078 | -0.04 |
| PI 36:4 | 135 | 0.306 | 0.378 | **0.048** | -0.072 | 0.428 | 0.413 | 0.488 | 0.015 |
| PI 36:3 | 136 | 0.761 | 0.622 | 0.124 | 0.139 | 0.752 | 0.667 | 0.366 | 0.085 |
| PI 36:2 | 137 | 0.443 | 0.497 | 0.34 | -0.054 | 0.428 | 0.443 | 0.115 | -0.015 |
| PI 36:1 | 138 | 0 | 0 | 0.243 | 0 | 0.019 | 0.01 | 0.228 | 0.009 |
| **Total PI** |  | 14.673 | 18.159 | 0.349 | -3.486 | 15.883 | 15.659 | 0.055 | 0.224 |
| LPG 16:1 | 139 | 0 | 0 | 0.165 | 0 | 0.029 | 0.118 | 0.27 | -0.089 |
| LPG 16:0 | 140 | 0.388 | 0 | **0.035** | 0.388 | 0.533 | 0.182 | 0.27 | 0.351 |
| LPG 18:3 | 141 | 0.185 | 0.149 | 0.151 | 0.036 | 0.552 | 0.102 | 0.455 | 0.45 |
| LPG 18:2 | 142 | 0 | 0.227 | 0.167 | -0.227 | 0 | 0.033 | 0.211 | -0.033 |
| LPG 18:1 | 143 | 0.619 | 0.501 | 0.179 | 0.118 | 0.091 | 0.192 | 0.237 | -0.101 |
| **Total LPG** |  | 1.192 | 0.877 | 0.1 | 0.315 | 1.2044 | 0.628 | 0.272 | 0.5764 |
| LPC(16:1) | 144 | 0 | 0 | 0.211 | 0 | 0 | 0 | 0.256 | 0 |
| LPC(16:0) | 145 | 0.018 | 0.021 | 0.452 | -0.003 | 0.034 | 0.02 | 0.126 | 0.014 |
| LPC(18:3) | 146 | 0.013 | 0.021 | 0.198 | -0.008 | 0.012 | 0.016 | 0.209 | -0.004 |
| LPC(18:2) | 147 | 0.013 | 0.016 | 0.368 | -0.003 | 0.015 | 0.014 | 0.172 | 0.001 |
| LPC(18:1) | 148 | 0 | 0.002 | 0.121 | -0.002 | 0.002 | 0.001 | **0.02** | 0.001 |
| LPC(18:0) | 149 | 0.005 | 0.007 | 0.086 | -0.002 | 0.010 | 0.003 | 0.177 | 0.007 |
| **TOTAL LPC** |  | 0.049 | 0.068 | 0.105 | -0.019 | 0.073 | 0.055 | 0.115 | 0.018 |
| LPE(16:1) | 150 | 0 | 0 | 0.246 | 0 | 0 | 0 | 0.178 | 0 |
| LPE(16:0) | 151 | 0.053 | 0.043 | 0.09 | 0.01 | 0.062 | 0.078 | 0.216 | -0.016 |
| LPE(18:3) | 152 | 0.025 | 0.031 | 0.383 | -0.006 | 0.034 | 0.035 | 0.495 | -0.001 |
| LPE(18:2) | 153 | 0.036 | 0.043 | **0.045** | -0.007 | 0.021 | 0.037 | 0.17 | -0.016 |
| LPE(18:1) | 154 | 0.002 | 0.004 | 0.357 | -0.002 | 0.003 | 0.001 | **0.034** | 0.002 |
| **TOTAL LPE** |  | 0.117 | 0.122 | 0.196 | -0.005 | 0.120 | 0.151 | 0.283 | -0.031 |
